# Glial Contributions to Pain in Neurodegenerative Disease Models: A Systematic Review of Microglial and Astrocytic Evidence

**DOI:** 10.3390/brainsci16101093

**Published:** 2026-10-10

**Authors:** Alfonso Alfaro-Rodríguez, José Luis Cortes-Altamirano, Samuel Reyes-Long, Herlinda Bonilla-Jaime, Francisco Jacob Avila-Camacho, Leonardo Miguel Moreno-Villalba, María Elizabeth Herrera-López, Angelica González-Maciel, Beatríz Pérez-Guille, Rosa Eugenia Soriano-Rosales, David Calderon-Guzman, Hugo Juaréz-Olguín

**Affiliations:** 1 División de Neurociencias Básicas, Instituto Nacional de Rehabilitación-LGII, Mexico City 14389, Mexico; alfa1360@yahoo.com.mx (A.A.-R.); sam.long27@gmail.com (S.R.-L.); 2 Laboratorio Nacional CONAHCyT en Inteligencia Artificial y Ciencia de Datos (LNC-IACD), Ecatepec de Morelos 55210, Mexico; 3 Departamento de Quiropráctica, Universidad Estatal del Valle de Ecatepec, Ecatepec de Morelos 55210, Mexico; 4 Departamento de Biología de la Reproducción, Universidad Autónoma Metropolitana-Iztapalapa, Mexico City 09340, Mexico; 5 División de Ingeniería en Sistemas Computacionales, TecNM/ Tecnológico de Estudios Superiores de Ecatepec, Ecatepec de Morelos 55210, Mexico; 6 División de Ingeniería Informática, TecNM/ Tecnológico de Estudios Superiores de Ecatepec, Ecatepec de Morelos 55210, Mexico; 7 Laboratorio de Morfología Celular y Tisular, Instituto Nacional de Pediatría, Secretaría de Salud, Mexico City 04300, Mexico; 8 Centro de Investigación Traslacional, Instituto Nacional de Pediatría, Mexico City 04300, Mexico; 9 Laboratorio de Neurociencias, Instituto Nacional de Pediatría, Mexico City 04530, Mexico; 10 Departamento de Farmacología, Facultad de Medicina, Universidad Nacional Autónoma de México, Mexico City 04510, Mexico

**Keywords:** neurodegeneration, chronic pain, microglial activation, astrocyte reactivity, neuroinflammation, nociceptive processing, glial modulation, central nervous system

## Abstract

**Background/Objectives:** Pain is a common and disabling non-motor manifestation of neurodegenerative disorders, but the contribution of glial cells to nociceptive processing remains incompletely understood. This systematic review evaluated evidence from preclinical models of neurodegenerative diseases linking microglial and astrocytic activity to pain. **Methods:** The protocol was prospectively registered in PROSPERO (CRD420251084310), and reporting followed PRISMA 2020. We conducted the original electronic search in PubMed/MEDLINE; during major revision, we conducted supplementary searches in Scopus and Web of Science Core Collection. Across the three sources, we identified 2248 records. After hierarchical deduplication by DOI, PMID, and normalized title, 1567 unique records were screened. Eligible in vivo studies assessed microglial and/or astrocytic alterations together with pain- or nociception-related outcomes. Risk of bias was independently evaluated by two investigators using the SYRCLE tool, and findings were synthesized qualitatively. **Results:** Twenty-six studies met the eligibility criteria: sixteen examined multiple sclerosis/experimental autoimmune encephalomyelitis (61.5%), seven Parkinson’s disease (26.9%), two Alzheimer’s disease-related models (7.7%), and one Huntington’s disease model (3.8%); no eligible amyotrophic lateral sclerosis study was identified. Pain-related alterations involved inflammatory, neuroimmune, synaptic, and astrocyte-associated pathways, but the evidence was strongly model-dependent. Findings based on individual studies, including early astrocytic contributions in experimental autoimmune encephalomyelitis and altered inflammatory allodynia in an Alzheimer’s disease model, are interpreted as study-specific observations requiring replication. **Conclusions:** The available evidence supports context-dependent associations between glial responses and nociceptive processing; however, marker specificity, EAE predominance, sex and age imbalance, and incomplete reporting of bias control procedures limit causal and cross-disease generalization.

## 1. Introduction

Pain is an increasingly recognized, albeit heterogeneous, manifestation of neurodegenerative disorders. Although motor, cognitive, and behavioral dysfunctions constitute defining clinical features of many of these diseases, pain symptoms may substantially contribute to disability and the reduction in quality of life. Pain is particularly relevant in Parkinson’s disease (PD) and Multiple Sclerosis (MS), and alterations in pain perception and chronic pain have also been described in Alzheimer’s disease (AD) and other neurodegenerative conditions [1,2,3]. Importantly, pain in these disorders is unlikely to result from a single disease-independent mechanism. In addition to peripheral and musculoskeletal processes, neurodegenerative pathology may alter central networks involved in sensory discrimination, descending pain control, affective processing, and nociceptive integration. Consequently, altered pain perception may arise from interactions between disease-specific neuronal pathology and changes in the neuroimmune microenvironment [4,5].

Neuroinflammation may bridge neurodegeneration and altered nociceptive processing. Within the central nervous system (CNS), microglia and astrocytes participate in tissue homeostasis and dynamically regulate neuronal function, synaptic activity, extracellular homeostasis, and immune responses [6,7]. Under pathological conditions, both cell types undergo transcriptional, morphological, and functional changes that may modify their interactions with neuronal circuits. Reactive glial states may influence the production or regulation of cytokines, chemokines, neurotrophic factors, damage-associated molecular patterns (DAMPs), and neurotransmitter-related pathways that can alter neuronal excitability [4,7]. These mechanisms are particularly relevant to pain because persistent neuroimmune signaling may facilitate central sensitization and maladaptive synaptic plasticity within spinal and supraspinal nociceptive networks [5,8]. However, glial reactivity is heterogeneous and context-dependent and should not be interpreted as an intrinsically pathological cellular state or a uniformly harmful process.

Microglia have received particular attention for their role in regulating pathological pain. Experimental evidence has implicated microglial inflammatory and neuron–glia signaling pathways involving pro-inflammatory cytokines, HMGB1, CX3CL1–CX3CR1 signaling, neurotrophic factors, and purinergic receptors in altered nociceptive transmission [3,4,5,9]. In experimental autoimmune encephalomyelitis (EAE), for example, increased CX3CL1/CX3CR1 expression in the spinal cord has been reported to accompany sensory abnormalities, with resident spinal microglia representing an important cellular source of CX3CR1 [9]. Similarly, in a hemiparkinsonian model, HMGB1 signaling was associated with mechanical hypersensitivity and spinal microglial activation [3]. However, in AD-related pathology, the relationship is not uniformly pronociceptive. Sideris-Lampretsas et al. [10] demonstrated that spinal microglial Galectin-3 signaling produced inflammatory nociception in wild-type mice but was altered in TASTPM mice modeling Alzheimer’s disease. These observations suggest that microglial involvement in nociception depends on the underlying disease environment rather than reflecting a universal relationship between microglial activation and pain.

Astroglial cells provide a complementary and less extensively integrated component of neurodegeneration-associated pain. Beyond their homeostatic functions, astrocytes regulate extracellular glutamate and ion concentrations, synaptic transmission, inflammatory signaling, and neuronal excitability, all of which are directly relevant to central sensitization. Evidence from neurodegenerative disease models indicates that astroglial responses may occur alongside microglial alterations but may also independently contribute to nociceptive dysfunction. In MS, Ding et al. [11] found an important role for spinal dorsal horn astrocytes in early pain, implicating astrocyte-dependent LRRC8A-mediated glutamate release and STAT3 signaling. Furthermore, genetic and pharmacological modulation of kinin B1/B2 receptors attenuated tactile and thermal hypersensitivity in MS, and B1 receptor expression was associated with astrocytes and reduced glial inflammatory signaling [12]. Other studies in MS have shown concurrent alterations in microglia and astrocytes associated with mechanical, cold, and spontaneous pain phenotypes, supporting disease stage-dependent and region-specific glial contributions [2,8]. Collectively, these findings challenge interpretations of pain based solely on the magnitude of glial activation and instead support research of distinct microglial and astrocytic responses within disease-, anatomical-, and time-specific contexts.

Despite growing evidence linking glial biology to nociceptive processing, the preclinical literature that specifically addresses pain in neurodegenerative disease models remains fragmented, encompassing different diseases, anatomical regions, behavioral paradigms, glial populations, and molecular pathways. Importantly, much of the information linking glia to chronic pain derives from peripheral nerve injury or inflammatory pain models rather than neurodegenerative disease models themselves [13,14]. This distinction is crucial because mechanisms identified in general neuropathic pain paradigms cannot be automatically assumed to operate similarly within a progressively neurodegenerating CNS. The heterogeneity observed in the eligible literature, spanning from microglial, astrocytic, inflammatory, neurotrophic, purinergic, to synaptic mechanisms, warrants systematic integration rather than narrative description alone. The eligible literature was expected to be heterogeneous because it spans distinct disease models, anatomical regions, behavioral paradigms, glial markers, and experimental interventions. Accordingly, this systematic review aimed to synthesize and critically evaluate preclinical evidence on how microglial and astrocytic alterations contribute to pain and nociceptive modulation in animal models of neurodegenerative diseases. Specifically, we sought to characterize the glial populations, anatomical regions, pain-related phenotypes, and molecular pathways involved; identify convergent and disease-specific mechanisms; and evaluate the methodological quality and risk of bias of the available evidence.

## 2. Materials and Methods

### 2.1. Protocol Registration

This systematic review evaluated preclinical evidence on the contribution of microglial and astrocytic alterations to pain and nociceptive modulation in animal models of neurodegenerative diseases. The protocol was prospectively registered in PROSPERO (CRD420251084310) on 29 July 2025 (https://www.crd.york.ac.uk/PROSPERO/view/CRD420251084310 (accessed on 7 October 2026)), and reporting followed the Preferred Reporting Items for Systematic Reviews and Meta-Analyses (PRISMA 2020) statement [15]. The registered protocol planned searches in PubMed/MEDLINE, Scopus, Web of Science, Embase, and the Cochrane Library. The review as originally implemented used PubMed/MEDLINE; during major revision, supplementary searches were conducted in Scopus and Web of Science Core Collection to increase database coverage and screening sensitivity. Embase and the Cochrane Library were not searched. This deviation from the registered search plan is reported transparently and was introduced during revision in response to peer-review concerns; eligibility definitions, disease scope, pain/nociception outcomes, and the requirement for glial evidence remained conceptually aligned with the registered review question.

### 2.2. Eligibility Criteria

Eligibility criteria were defined a priori according to the registered protocol and were structured around the animal population, neurodegenerative disease model, glial exposure or intervention, and pain-related outcomes.

Studies were considered eligible if they met all of the following criteria: (1) original, peer-reviewed experimental research using an in vivo animal model; (2) investigation of a neurodegenerative disease or an established experimental model reproducing relevant neurodegenerative pathology, including Parkinson’s disease, Alzheimer’s disease, multiple sclerosis/experimental autoimmune encephalomyelitis, amyotrophic lateral sclerosis, Huntington’s disease (HD), or another clearly defined neurodegenerative disorder; (3) assessment of microglial and/or astrocytic alterations, including cellular reactivity, morphology, glial marker expression, inflammatory mediators, signaling pathways, or experimental modulation of glial function; and (4) assessment of at least one pain- or nociceptive-related outcome and an explicit relationship between the glial alteration and nociceptive processing. The registered eligibility criteria specifically required studies to report both glial alterations and pain-related outcomes.

Eligible pain-related outcomes included mechanical allodynia or hyperalgesia, thermal hyperalgesia, cold allodynia or cold hyperalgesia, spontaneous pain-related behaviors, and other experimentally validated measures of nociceptive processing. Neurophysiological or molecular correlates of nociception were considered supplementary outcomes when accompanied by a behavioral or other explicit pain-related phenotype.

Studies were excluded if they (1) were conducted exclusively in humans or in vitro; (2) investigated pain models without an underlying neurodegenerative disease model, including peripheral nerve injury, isolated inflammatory pain, cancer pain, or postoperative pain; (3) did not assess a pain- or nociception-related outcome; (4) did not assess microglia or astrocytes; (5) reported glial alterations without establishing an explicit relationship with nociceptive modulation; or (6) were reviews, editorials, letters, case reports, conference abstracts, non-peer-reviewed preprints, or other reports that were not full-length peer-reviewed original in vivo research. Studies published in languages other than English or Spanish were also excluded.

### 2.3. Information Sources and Literature Search

The original electronic search was conducted in PubMed/MEDLINE for publications from 1 January 2000, through 24 August 2026 and yielded 437 records. During major revision, supplementary searches were conducted on 25 September 2026 in Scopus (*n* = 1381) and Web of Science Core Collection (*n* = 430), using database-adapted combinations of glial, pain/nociception, and neurodegenerative disease concepts. Across the three sources, 2248 records were identified. Hierarchical deduplication using DOI, PMID, and normalized title removed 681 duplicate records, leaving 1567 unique records. Complete Scopus and Web of Science search strings, search dates, platforms, and retrieval counts are provided in Supplementary Table S1. The exact historical PubMed query was not preserved in the original export and was therefore not retrospectively reconstructed as if it were the original query.

Search concepts included microglia and astrocytes; pain and nociception (including allodynia, hyperalgesia, hypersensitivity, and related behavioral outcomes); and neurodegenerative diseases/models including Parkinson’s disease, Alzheimer’s disease, multiple sclerosis/EAE, amyotrophic lateral sclerosis, and Huntington’s disease. Boolean operators and database-specific field syntax were adapted to each source. No language restriction was applied at electronic retrieval; we applied English/Spanish eligibility during study selection.

### 2.4. Study Selection

For the original PubMed workflow, records underwent sequential title triage, title/abstract assessment, and full-text eligibility assessment according to the prespecified criteria. Two reviewers performed study selection independently, with disagreements resolved through discussion and consensus and, when necessary, consultation with a third reviewer. The preserved original screening workbook documented 437 records, 389 exclusions at initial title triage, 48 records retained for further assessment, 27 subsequent exclusions, and 21 full-text reports assessed and included. Because title-only triage may miss secondary glial outcomes, we screened revision-stage Scopus and Web of Science records by title and abstract before full-text assessment.

For the updated search, records from PubMed/MEDLINE, Scopus, and Web of Science were deduplicated hierarchically by DOI, PMID, and normalized title. Of 2248 raw records, 681 duplicates were removed and 1567 unique records were screened. A total of 1541 records were excluded before full-text retrieval; 26 reports were sought, all 26 were retrieved and assessed in full text, and none was excluded at the full-text stage. Five additional eligible studies were identified during the update, resulting in 26 studies in the final qualitative synthesis.

For the preserved original PubMed second-stage screening, the 27 exclusions were classified as follows: 13 review/bibliometric/non-original publications; 9 records without an eligible microglial/astrocytic outcome explicitly linked to pain or nociception; 2 records with an ineligible model/direction of the disease-pain relationship; 1 non-peer-reviewed preprint/ineligible publication type; 1 animal pain study without demonstration of the required glial-pain relationship in the animal component; and 1 record that did not clearly satisfy the combined glial + pain + neurodegenerative model eligibility criteria. Individual reasons were not retrospectively assigned to all 1541 records excluded during the updated title/abstract screening because those record-level reasons were not prospectively preserved.

**Figure 1:**
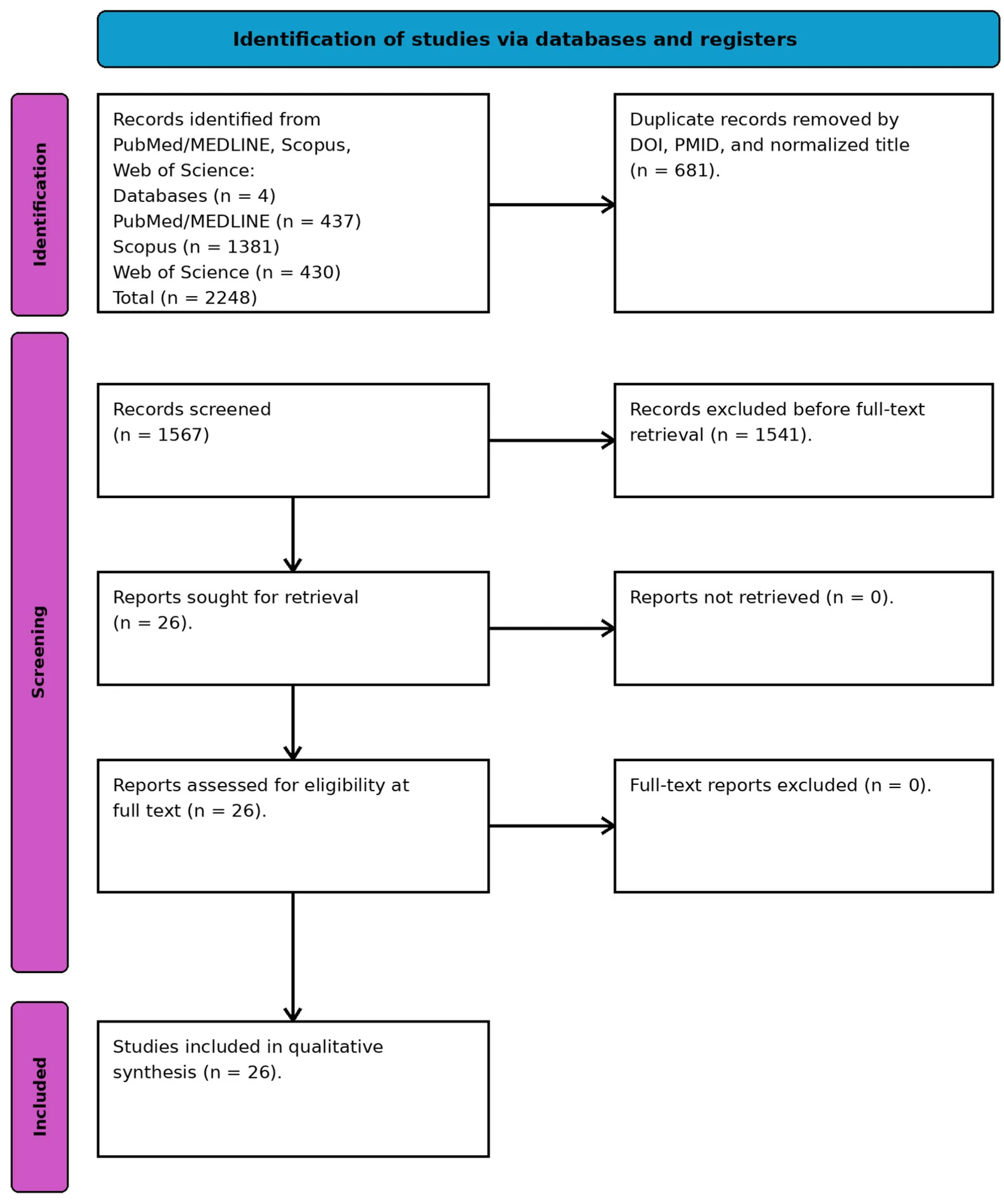
PRISMA 2020 flow diagram of study identification, screening, eligibility assessment and inclusion.

The numbers of records identified, removed as duplicates, screened, excluded, sought for retrieval, retrieved, assessed at full text, and included are reported in the revised PRISMA 2020 flow diagram (Figure 1). Pre-consensus reviewer decisions from the historical screening were not preserved in a form permitting a valid retrospective Cohen’s kappa calculation; therefore, no kappa coefficient was reconstructed post hoc.

### 2.5. Data Extraction

Two reviewers performed data extraction independently using a standardized extraction framework based on the variables prespecified in the PROSPERO protocol. Reviewers compared extracted data and resolved disagreements through discussion and consensus. A third reviewer resolved any remaining disagreements.

The following information was extracted from each eligible study: (a) bibliographic characteristics, including first author, year of publication, journal, and country; (b) experimental characteristics, including species, strain, sex, sample size, neurodegenerative disease model, and method of disease induction; (c) experimental groups and comparator conditions; (d) intervention or experimental manipulation; (e) anatomical region investigated; (f) microglial and/or astrocytic populations evaluated; (g) cellular markers and molecular pathways; (h) pain-related behavioral tests and associated phenotypes; (i) timing of assessment relative to disease induction or progression; and (j) the main glial and nociceptive findings.

The following information was extracted from each eligible study: (a) bibliographic characteristics; (b) species, strain, sex, age and/or body weight when reported, sample size, disease model, and induction method; (c) experimental groups and comparator conditions; (d) intervention or experimental manipulation; (e) anatomical region; (f) glial population and the marker(s) used to infer cell identity/state; (g) molecular pathways; (h) pain-related behavioral tests and the specific phenotype measured; (i) timing of glial and behavioral assessment relative to disease induction/progression; (j) effects on lesion severity, EAE clinical score, or motor performance when reported; and (k) the principal glial and nociceptive findings. Information absent from the primary report was recorded as not reported or unclear rather than inferred.

Primary outcomes included behavioral measures of pain or altered nociceptive sensitivity, such as mechanical allodynia or hyperalgesia, thermal hypersensitivity, cold allodynia or hyperalgesia, and spontaneous pain-related behaviors. Secondary outcomes included microglial and astrocytic markers, inflammatory mediators, signaling pathways, markers of neuronal activity, and other molecular or neurophysiological variables related to nociceptive processing.

When relevant information was absent or could not be verified from the available report, it was recorded as “not reported” or “unclear” rather than inferred.

To avoid reducing heterogeneous glial responses to a binary activated/nonactivated classification, findings were extracted according to the specific cellular and molecular evidence reported. In inflammatory models such as EAE, IBA1 or CD11b immunoreactivity alone was not considered sufficient to distinguish resident microglia from infiltrating macrophages/myeloid cells; where cellular specificity was unresolved, the synthesis uses terms such as microglia/macrophage-associated or myeloid/glial reactivity. Greater cellular specificity was attributed only when studies used markers or approaches capable of supporting resident microglial identity, such as TMEM119/P2RY12 or cell-specific manipulation. Likewise, increased GFAP was interpreted as astrocytic reactivity and not, by itself, as evidence of a functionally defined astrocyte state. DRG-level findings were described according to demonstrated cellular localization and were not treated as direct evidence of spinal microglial or astrocytic activation.

To avoid reducing heterogeneous glial responses to a binary activated/nonactivated classification, we extracted findings based on the specific cellular and molecular evidence reported in each study. Microglial and astrocytic involvement was therefore characterized using reported markers, morphology, molecular signaling, inflammatory mediators, cellular manipulation, and functional associations with nociceptive outcomes.

Terms such as “M1/M2 microglial polarization” or analogous binary classifications of reactive states were retained only when the original investigators used them explicitly. We did not impose such terminology as a standardized classification across studies.

For qualitative integration, molecular findings were organized around recurring functional mechanisms identified in the included literature, including neuroinflammatory amplification, neuroglial and synaptic signaling, central sensitization, and context-dependent glial regulation. These categories served as an interpretive framework for the synthesis, not as additional eligibility criteria.

### 2.6. Risk-of-Bias Assessment

Methodological quality and risk of bias were independently assessed by two investigators using the Systematic Review Center for Laboratory Animal Experimentation Risk of Bias tool (SYRCLE RoB tool) [16], as specified in the registered protocol. Disagreements were resolved through discussion and consensus, with consultation of a third investigator when necessary.

We evaluated the following domains: sequence generation, baseline characteristics, allocation concealment, random housing, blinding of caregivers and/or investigators, random outcome assessment, blinding of outcome assessors, incomplete data, selective outcome reporting, and other potential sources of bias.

Each domain was classified as having a low, high, or unclear risk of bias. Low risk was assigned only when the full report provided sufficient methodological information to support adequate control of bias; absence or insufficiency of reporting was classified as unclear rather than low risk. Particular attention was paid to attrition/humane-endpoint exclusions in EAE studies, selection of successfully lesioned animals in PD models, sequence generation, allocation concealment, random housing, and blinding. Detailed study-level judgments are provided in the Supplementary Materials, while the main text presents a color-coded summary figure.

### 2.7. Data Synthesis

Because of substantial heterogeneity in neurodegenerative disease models, animal species and strains, experimental interventions, anatomical regions, glial outcomes, molecular pathways, pain paradigms, behavioral measures, and reporting formats, quantitative meta-analysis was not considered appropriate. The findings were therefore synthesized qualitatively.

Studies were initially grouped by disease model and then compared by glial population, anatomical region, nociceptive phenotype, experimental intervention, and molecular mechanism. We paid particular attention to whether the findings supported predominantly microglial, predominantly astrocytic, or combined glial contributions, and whether these relationships varied by disease stage or anatomical context.

When appropriate, we integrated findings into recurring mechanistic modules rather than grouping them solely by individual molecular targets. This approach was intended to identify convergence among neuroinflammatory, neuroimmune, and synaptic mechanisms while preserving disease-specific differences.

The registered protocol anticipated qualitative subgroup analyses according to disease model, glial cell type, intervention or exposure, pain outcome, animal species or strain, and timing relative to disease induction.

## 3. Results

### 3.1. Study Selection

The updated searches identified 2248 records: 437 from PubMed/MEDLINE, 1381 from Scopus, and 430 from Web of Science Core Collection. Hierarchical deduplication by DOI, PMID, and normalized title removed 681 duplicates, leaving 1567 unique records for screening.

A total of 1541 records were excluded before full-text retrieval. Twenty-six reports were sought for retrieval; all 26 were obtained and assessed for eligibility at full text, and none was excluded at that stage. The revision-stage search identified five additional eligible studies beyond the 21 studies in the original PubMed-based synthesis, resulting in 26 studies in the final qualitative synthesis.

The original PubMed screening workbook additionally permitted reconstruction of the 27 second-stage exclusions: 13 review/bibliometric/non-original reports, 9 without an eligible glial outcome linked to pain/nociception, 2 with an ineligible model/direction, 1 non-peer-reviewed preprint/ineligible publication type, 1 without demonstration of the required animal glial-pain relationship, and 1 that did not clearly meet the combined eligibility criteria. The complete updated selection process is summarized in Figure 1.

### 3.2. General Characteristics of Included Studies

The 26 included studies comprised preclinical investigations examining pain- or nociception-related outcomes in animal models of neurodegenerative diseases in relation to microglial and/or astrocytic alterations. The evidence base remained markedly uneven: sixteen studies (61.5%) investigated MS/EAE, seven (26.9%) investigated PD, two (7.7%) addressed AD-related models, and one (3.8%) investigated HD. No eligible ALS study met all inclusion criteria. Thus, 23 of 26 studies (88.5%) were derived from MS/EAE or PD models, which limits cross-disease generalization.

Rodents were used throughout the evidence base. Female mice predominated in EAE studies, whereas PD studies included both mice and rats. Age was inconsistently reported and most experiments used young or young-adult animals, an important limitation for age-associated neurodegenerative disorders. EAE was commonly induced by myelin antigen immunization and is interpreted here as an immune-mediated demyelinating model with secondary neurodegenerative processes rather than as equivalent to primarily neurodegenerative models. PD studies predominantly used 6-hydroxydopamine (6-OHDA) or MPTP. Evidence for AD and HD was derived from transgenic models.

Rodents were employed throughout the included evidence base, with mice predominating in EAE studies and both mice and rats being used in PD models. EAE was commonly induced using immunization paradigms involving myelin oligodendrocyte glycoprotein, whereas PD studies primarily used dopaminergic neurodegeneration induced by 6-hydroxydopamine (6-OHDA) or 1-methyl-4-phenyl-1,2,3,6-tetrahydropyridine (MPTP). Evidence for AD was derived from transgenic models, whereas the single HD study used a transgenic model of Huntington’s disease.

Pain-related phenotypes included mechanical allodynia or hypersensitivity, mechanical hyperalgesia, thermal hyperalgesia or altered thermal sensitivity, cold hypersensitivity, spontaneous pain-related behavior, trigeminal nociception, and altered analgesic responses. Generic terms such as “hypernociception” were replaced, wherever the primary study permitted, by the specific modality and assay reported. Table 1 summarizes study characteristics, including strain, sex, age/body weight when available, glial markers, and the specific nociceptive phenotype.

### 3.3. Glial Populations and Anatomical Distribution

Most included studies investigated microglial/myeloid alterations, either independently or alongside astrocytic responses. Interpretation depended on marker specificity. In EAE, IBA1 and CD11b can label resident microglia as well as infiltrating macrophage/myeloid populations; therefore, studies relying on these markers alone are described conservatively as showing microglia/macrophage-associated or myeloid/glial reactivity. By contrast, TMEM119/P2RY12 or cell-specific manipulations provide stronger support for resident microglial identity. GFAP elevation is described as astrocytic reactivity rather than a functionally defined reactive astrocyte state.

The spinal dorsal horn was among the most frequently investigated regions, particularly in EAE studies. Other regions included dorsal root ganglia (DRG), striatum, prefrontal/anterior cingulate regions, central amygdala, and other CNS structures. DRG findings were retained only as tissue- or cell-specific evidence supported by the source study; DRG expression was not interpreted as direct evidence of spinal microglial or astrocytic activation because the DRG contains sensory neurons, satellite glial cells, and macrophage populations rather than CNS microglia and astrocytes.

For example, Zhu et al. [9] reported CX3CL1/CX3CR1 changes in DRG and spinal cord associated with sensory abnormalities. In the revised synthesis, the cellular source is reported only where demonstrated by localization data, and CX3CL1 is not assigned uniformly to a single cell type across tissues. This resolves the previous inconsistency between descriptions of neuronal/astrocytic and predominantly neuronal CX3CL1 expression.

Importantly, microglial and astrocytic responses were not always synchronous. Ding et al. [11] demonstrated prominent astrocytic reactivity in the dorsal gray matter of the spinal cord during the preclinical stage of EAE and reported that inhibition of astrocytic reactivity attenuated early pain-related behavior, whereas spinal microglia did not appear to contribute directly during this initial phase. These findings indicate that cellular contributions to nociceptive alterations vary by anatomical region and temporal stage, rather than reflecting uniform, parallel activation of microglia and astrocytes.

### 3.4. Multiple Sclerosis and Experimental Autoimmune Encephalomyelitis

MS/EAE represented the largest disease-specific evidence group, accounting for 16 of 26 included studies (61.5%). Because EAE is an induced autoimmune-inflammatory model with lymphocyte and monocyte/macrophage infiltration, recurring inflammatory and glial findings in this group are interpreted primarily as MS/EAE-specific evidence unless reproduced in other disease models.

#### 3.4.1. Astrocytic Contribution to Early-Stage EAE-Associated Pain

Ding et al. [11] provided a single-study example of temporally differentiated glial involvement. In that EAE experiment, spinal dorsal-horn astrocytic reactivity was detected during the preclinical stage and astrocyte-directed manipulation attenuated early pain-related behavior, whereas prominent microglial involvement was not demonstrated at that time point. This observation is therefore treated as study-specific evidence requiring independent replication rather than as a general principle across neurodegenerative diseases.

Mechanistic analyses suggested that LRRC8A-mediated glutamate release may contribute to STAT3 activation and astrocytic reactivity, thereby linking disrupted astrocytic homeostasis to early EAE-associated nociception.

Complementary evidence from Dutra et al. [12] showed that kinin B1 and B2 receptors are involved in persistent tactile and thermal hypersensitivity during EAE, and that astrocytic signaling contributes to the observed nociceptive phenotype.

Collectively, these studies provide evidence that astrocytes can contribute functionally to EAE-associated pain rather than representing only a secondary component of microglial activation.

#### 3.4.2. Microglial Inflammatory Signaling in EAE

Microglial alterations were also repeatedly associated with nociceptive dysfunction in EAE. Serizawa et al. [18] investigated interleukin-6 (IL-6) signaling and demonstrated that IL-6 receptor inhibition modified pain-related behavior in EAE while also affecting inflammatory and glial responses in the spinal cord. The study specifically evaluated the relationship between clinical manifestations of the disease and analgesic effects following treatment with an IL-6 receptor inhibitor.

Zhu et al. [9] identified increased expression of the CX3CL1–CX3CR1 axis in the dorsal root ganglia and spinal cord, with molecular alterations correlating with behavioral evidence of altered nociception.

These observations support an association between microglial neuroimmune signaling and altered nociceptive processing during EAE, although the strength of the causal inference varied among studies depending on whether the molecular pathway had been experimentally manipulated.

#### 3.4.3. Central Sensitization and S1PR1 Signaling

Doolen et al. [8] provided evidence linking sphingosine-1-phosphate receptor 1 (S1PR1) signaling to EAE-associated hypersensitivity and central sensitization. Fingolimod reduced mechanical and cold hypersensitivity in a dose-dependent and reversible manner. Importantly, its analgesic effects occurred before any significant improvement in motor disability, providing temporal evidence that the reduction in pain-related behavior was not merely secondary to improved neurological function. The study also evaluated indices of central sensitization in the dorsal horn, including neuronal calcium signaling, ERK phosphorylation, GFAP, and Iba1, and related the behavioral improvement to changes in neuronal and glial responses.

#### 3.4.4. Combined Microglial and Astrocytic Responses Involving TRPA1

Dalenogare et al. [25] evaluated the contribution of the Transient Receptor Potential Ankyrin 1 (TRPA1) to EAE and reported that genetic deletion of TRPA1 attenuated pain-related hypersensitivity and neuroinflammatory responses. Changes in both microglial and astrocytic markers support the involvement of combined glial responses in the nociceptive phenotype.

These results extend the EAE evidence beyond canonical cytokine signaling and suggest that ion channel-dependent mechanisms may modulate both sensory dysfunction and glial reactivity.

#### 3.4.5. Disease Severity and Temporal Evolution

Disease severity and stage strongly influenced the relationship between EAE and pain. Segal et al. [20] showed that EAE severity and neuroinflammation regulated chronic mechanical hypersensitivity. This observation is methodologically important because severe motor dysfunction may interfere with withdrawal-based nociceptive testing and potentially mask, rather than eliminate, pain-related phenotypes.

Across longitudinal EAE investigations, nociceptive behaviors, including mechanical, cold, spontaneous, and other forms of sensitivity, varied throughout disease progression alongside changes in microglial and astrocytic responses. The collective evidence therefore indicates that glial cells’ contribution to EAE-associated pain is dynamic over time.

### 3.5. Parkinson’s Disease

Seven studies investigated glial evidence associated with altered nociception in PD models. Most used unilateral 6-OHDA lesions, whereas one used MPTP and the updated evidence also included noradrenergic modulation in a 6-OHDA rat model. The PD literature predominantly identified mechanical and/or thermal hypersensitivity associated with glial and neuroinflammatory changes at spinal or supraspinal levels; these phenotypes are not automatically classified as neuropathic pain unless justified by the primary study.

#### 3.5.1. HMGB1 and Spinal Microglial Signaling

Sato et al. [3] demonstrated that High-Mobility Group Box 1 (HMGB1) signaling contributed to mechanical hypersensitivity in a hemiparkinsonian model. Pain-related behavior was accompanied by spinal microglial activation, and HMGB1 neutralization attenuated both hypersensitivity and microglial responses.

A key feature of this study was that pain-related behavior was modulated without reversal of the underlying dopaminergic lesion, supporting a partial dissociation between the primary neurodegenerative lesion and downstream neuroimmune mechanisms regulating nociception.

#### 3.5.2. Microglial and Astrocytic Reactivity and NF-κB Signaling

Godínez-Chaparro et al. [27] investigated the effects of pramipexole in rats with 6-OHDA-induced lesions. The study used behavioral validation of the dopaminergic lesion and subsequently evaluated nociceptive outcomes and histological findings. Researchers selected animals based on established motor and lesion criteria, including apomorphine-induced rotational behavior.

Pramipexole attenuated mechanical pain-related alterations and was associated with reduced microglial and astrocytic reactivity. NF-κB/p65-related signaling in astrocytes was among the molecular responses associated with treatment, suggesting that dopaminergic pharmacotherapy may influence nociception partly through neuroimmune mechanisms.

#### 3.5.3. Glial Responses in the Striatum

Zhang et al. [26] reported that a unilateral 6-OHDA-induced dopaminergic lesion was accompanied by mechanical allodynia and altered microglial and astrocytic profiles in the striatum. Minocycline treatment attenuated both pain-related behavior and inflammatory glial responses.

These observations extend the anatomical distribution of pain-associated glial alterations beyond the spinal cord and suggest that supraspinal structures affected by the neurodegenerative process may participate in altered nociceptive processing.

#### 3.5.4. NLRP3 Inflammasome Signaling

Wang et al. [30] identified an association among nociceptive hypersensitivity, microglial reactivity, and activation of the NLRP3–ASC–caspase-1 inflammasome pathway in an MPTP model. Electroacupuncture increased nociceptive thresholds while reducing microglial activation, inflammasome signaling, and inflammatory mediators, including IL-1β, IL-6, and TNF-α.

These findings provide experimental support for inflammasome-associated neuroimmune signaling as a mechanism contributing to altered nociception during dopaminergic neurodegeneration.

#### 3.5.5. Descending Monoaminergic and Cav2.2-Related Regulation

Evidence from PD models also implicated descending monoaminergic control. Campos et al. [22] identified alterations involving descending serotonergic modulation and spinal astrocytic responses in a 6-OHDA model.

Cui et al. [29] further reported that α2-adrenergic receptor modulation influenced Cav2.2-mediated nociceptive signaling in a 6-OHDA/DSP-4 Parkinsonian rat model and was associated with GFAP-positive astrocytic and IBA1-positive microglia/macrophage-associated responses in the spinal dorsal horn. The complete full text was retrieved and re-evaluated for eligibility, extraction, and risk-of-bias assessment; unreported methodological domains were classified as unclear rather than inferred.

### 3.6. Alzheimer’s Disease

Only two studies met the eligibility criteria for AD, making this evidence considerably more limited than that available for EAE or PD.

Sideris-Lampretsas et al. [10] investigated inflammatory allodynia in TASTPM mice and identified altered spinal microglial signaling involving Galectin-3, TLR4, P2Y12, and p38. Importantly, the AD model did not simply show increased pain alongside increased microglial reactivity. Instead, TASTPM animals exhibited attenuated inflammatory allodynia accompanied by an altered microglial response.

Sideris-Lampretsas et al. [10] investigated inflammatory mechanical allodynia in TASTPM mice and identified altered spinal microglial signaling involving Galectin-3, TLR4, P2Y12, and p38. This single study reported attenuated inflammatory allodynia together with an altered microglial response in the AD model. The finding is therefore presented as a model-specific observation and not as evidence that advanced Alzheimer’s pathology generally reduces pain.

Jin et al. [19] investigated functional modulation of microglia in a transgenic tauopathy model. Experimental manipulation of microglial signaling was associated with changes in pain-related behavior, further supporting the idea that microglial state may influence nociceptive processing in AD-related pathology.

Nevertheless, because only two eligible studies addressed AD, the evidence is insufficient to establish a broadly generalizable, AD-specific glial mechanism of pain.

### 3.7. Huntington’s Disease

Only one eligible study investigated HD. The study reported altered nociceptive and analgesic responses in a transgenic HD model, along with changes in microglial and astrocytic inflammatory profiles in the spinal cord. Alterations in thermal nociception and responses to opioid analgesics were accompanied by changes in selected inflammatory mediators. Because this evidence came from a single study, these observations should be regarded as preliminary, disease-specific evidence rather than evidence of a reproducible HD-associated glial mechanism.

### 3.8. Cross-Disease Mechanistic Synthesis

Despite substantial heterogeneity among disease models, anatomical regions, behavioral paradigms, and experimental interventions, comparison across studies suggested recurring mechanistic patterns linking glial alterations to nociceptive processing. Rather than converging on a single molecular pathway or a uniform state of glial activation, the evidence could be organized into four partially overlapping functional modules: (I) neuroinflammatory amplification, (II) neuroglial and synaptic signaling, (III) astrocyte-dependent and multimodal glial regulation, and (IV) context-dependent glial regulation (Table 2).

The first module comprised inflammatory mechanisms involving the NLRP3–ASC–caspase-1 inflammasome, IL-1β, IL-6, TNF-α, HMGB1, and NF-κB-related signaling. These pathways were identified primarily in EAE and Parkinsonian models and were generally associated with nociceptive hypersensitivity and reactive glial responses. Experimental attenuation of selected inflammatory pathways was accompanied by reductions in pain-related behavior and/or glial inflammatory markers [3,18,27,30].

The second module encompassed mechanisms linking glial responses to neuronal excitability, synaptic plasticity, and descending nociceptive control. These included CX3CL1–CX3CR1, BDNF–TrkB–ERK, S1PR1-dependent signaling, descending serotonergic modulation, and α2-adrenergic receptor–Cav2.2 signaling [8,9,17,22,29]. Collectively, these studies indicate that glial involvement in neurodegeneration-associated pain extends beyond the release of inflammatory mediators and includes modulation of neuronal and synaptic processes involved in central sensitization. 

A third pattern involved astrocyte-dependent or multimodal glial mechanisms. Dutra et al. [12] implicated kinin B1/B2 receptor signaling in persistent EAE-associated pain, whereas Ding et al. [11] identified an astrocytic LRRC8A–STAT3-related mechanism during early EAE-associated hypersensitivity. Importantly, the latter study demonstrated a functional astrocytic contribution at a disease stage in which prominent microglial involvement was not evident. In contrast, Dalenogare et al. [25] identified TRPA1-dependent modulation involving both microglial and astrocytic neuroinflammatory responses. Collectively, these findings argue against an obligatory sequential model in which microglial activation must precede astrocytic involvement.

Finally, the fourth module reflected the context dependence of glial regulation of nociception. Glial contributions varied according to disease model, anatomical region, disease stage or severity, and, in selected studies, sex. Particularly informative was the AD-related evidence showing that disease-associated microglial alterations were accompanied by attenuated rather than intensified inflammatory allodynia [10]. Similarly, longitudinal EAE studies demonstrated that the relationship between pain-related behavior and glial alterations varied with disease progression and severity [2,20], whereas Dworsky-Fried et al. [24] identified region- and sex-dependent neuroimmune alterations within the central amygdala.

Overall, the cross-disease synthesis indicates that the available evidence does not support a simple linear relationship between increased glial reactivity and increased pain. Instead, microglial and astrocytic contributions appear to depend on the molecular pathway involved, the anatomical compartment, the underlying neurodegenerative disease, and the temporal stage of the pathology.

### 3.9. Risk of Bias

**Figure 2:**
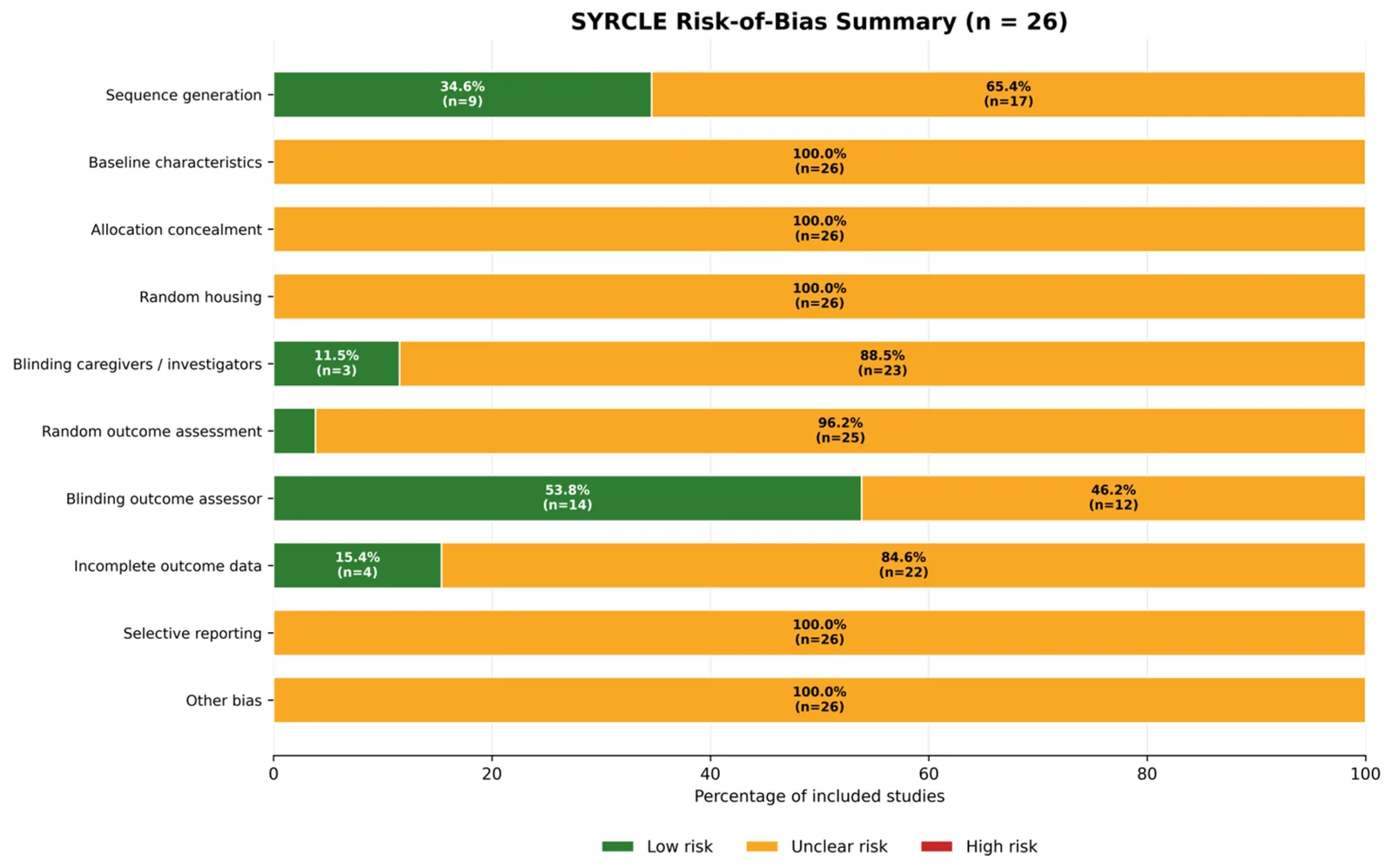
Risk-of-bias assessment of the included animal studies using the SYRCLE tool.

We assessed risk of bias in all 26 included studies using the SYRCLE tool (Figure 2). The predominant classification across the evidence base was unclear risk of bias, mainly because methodological parameters were incompletely reported and clearly high-risk procedures were not identified.

A subset of studies explicitly reported random sequence generation, including studies in which animals were randomly allocated to experimental groups. For example, Teixeira et al. [21] reported random allocation by drawing lots. Other studies provided explicit evidence of blinded behavioral assessment, histological analysis, or both.

However, allocation concealment, random housing, blinding of caregivers or investigators, and random outcome assessment were frequently insufficiently described. In accordance with the predefined assessment strategy, we classified the absence of methodological reporting as unclear rather than automatically as a high risk of bias. Overall, incomplete reporting in experimental studies limited our certainty about methodological bias across the evidence base.

## 4. Discussion

### 4.1. Main Findings

This systematic review qualitatively synthesized 26 preclinical studies examining microglial and/or astrocytic evidence together with pain- or nociception-related outcomes in neurodegenerative disease models. The updated evidence base was dominated by MS/EAE (16 studies; 61.5%) and PD (7 studies; 26.9%), with only two AD-related studies and one HD study. Accordingly, apparent cross-disease convergence must be interpreted cautiously: recurrent inflammatory or glial signatures may partly reflect the predominance of EAE, an immune-mediated model in which neuroinflammation and myeloid cell infiltration are intrinsic features of disease induction.

Across studies, glial reactivity frequently co-occurred with altered nociceptive behavior and, in some intervention studies, behavioral improvement accompanied changes in glial or inflammatory measures. These parallel changes support biological association but do not, by themselves, establish cell-specific causality. The strength of inference was greater when studies used cell-selective markers/manipulations, temporal dissociation, or interventions that altered nociception without simultaneously improving the primary disease phenotype.

Despite this imbalance, several convergent biological processes emerged. Microglial and astrocytic contributions extended beyond the release of conventional inflammatory mediators and included neuroimmune signaling, modulation of synaptic transmission, ion-channel regulation, and descending monoaminergic control. These findings could be organized into four interconnected functional modules: neuroinflammatory amplification, neuroglial and synaptic signaling, astrocyte-dependent glial regulation, and context-dependent glial regulation. Importantly, these modules should not be interpreted as independent pathways. Rather, they represent interacting components of a dynamic neuroglial network that can modify neuronal excitability and nociceptive processing at spinal and supraspinal levels.

Collectively, our findings support a model in which pain-related glial responses are determined by the functional state of microglia and astrocytes within a disease- and region-specific environment, rather than by generalized glial activation. This distinction matters because several included studies showed experimental modulation of pain-related behavior through specific glial pathways, while others revealed temporal, regional, or disease-dependent dissociations between glial reactivity and nociceptive outcomes. Accordingly, the available evidence suggests that microglia and astrocytes act as context-sensitive regulators of nociceptive processing during neurodegeneration.

### 4.2. Neuroinflammatory Amplification as a Shared but Non-Universal Mechanism

One of the most consistent patterns across the included studies was the association between pain-related behavior and neuroinflammatory signaling involving microglia and/or astrocytes. However, rather than a single inflammatory cascade shared by all neurodegenerative disorders, the evidence points to convergence among several partially interconnected pathways, including IL-6/IL-6R, HMGB1, NF-κB, and the NLRP3–ASC–caspase-1 inflammasome, along with downstream inflammatory mediators such as IL-1β and TNF-α. Experimental modulation of these pathways was frequently accompanied by changes in nociceptive behavior, supporting neuroinflammation as an important mechanism that amplifies altered pain processing.

Evidence from EAE models was particularly informative. Serizawa et al. [18] demonstrated that blockade of IL-6 signaling with an anti-IL-6 receptor antibody reduced mechanical pain hypersensitivity and attenuated the increase in spinal microglial reactivity in mice with EAE. Importantly, the treatment remained effective after pain hypersensitivity had developed, supporting an association between sustained IL-6-dependent neuroimmune signaling and maintenance of the nociceptive phenotype rather than an exclusively disease-initiating effect. Together with other EAE studies demonstrating relationships among spinal neuroinflammation, glial responses, and mechanical hypersensitivity [2,20], these findings indicate that inflammatory signaling may contribute to both the development and persistence of pain during autoimmune neurodegeneration.

A related neuroimmune mechanism emerged from Parkinsonian models. Sato et al. [3] identified HMGB1 signaling in association with mechanical hypersensitivity and spinal microglial responses in hemiparkinsonian mice. HMGB1 is particularly relevant because, as a damage-associated molecular pattern, it may provide a molecular interface between neuronal injury and innate immune responses. Within this framework, neurodegeneration-associated neuronal damage may generate extracellular signals that modify microglial function, which may in turn alter the inflammatory environment surrounding nociceptive circuits. The findings of Sato et al. therefore support the hypothesis that pain in neurodegenerative diseases may arise partly from bidirectional interactions between neuronal pathology and neuroimmune signaling rather than exclusively from neuronal dysfunction.

The NLRP3 inflammasome represents another important component of this inflammatory network. In an MPTP model of Parkinson’s disease, Wang et al. [30] reported that electroacupuncture-mediated attenuation of pain-related behavior was associated with inhibition of the microglial NLRP3–ASC inflammatory pathway in the amygdala. This observation is consistent with broader evidence implicating NLRP3 activation in both neurodegenerative and chronic pain mechanisms. Assembly of the NLRP3 inflammasome promotes caspase-1-dependent maturation of IL-1β and IL-18, thereby providing a biologically plausible mechanism through which innate immune signaling may influence neuronal excitability, synaptic plasticity, and central sensitization. However, this systematic review supports this pathway only in selected experimental settings. NLRP3 should therefore not be regarded as a universal mechanism underlying pain across all neurodegenerative diseases.

Astrocyte-associated inflammatory signaling further expands this framework. Godínez-Chaparro et al. [27] demonstrated that pramipexole reduced allodynia and hyperpathia in a rat model of PD in association with modulation of NF-κB-related signaling in astrocytes. This finding matters because it shows that neuroinflammatory modulation of nociception cannot be attributed exclusively to microglia. NF-κB acts as a major transcriptional regulator of inflammatory responses and may link extracellular inflammatory signals to changes in cytokine expression and astrocytic function. Microglial and astrocytic inflammatory responses may therefore converge on overlapping molecular pathways while exerting distinct effects depending on the cellular and anatomical context.

Interactions among these pathways also provide a plausible mechanism for amplification of nociceptive signaling. IL-1β, IL-6, TNF-α, HMGB1, and inflammasome-associated signaling may modify the extracellular environment of nociceptive circuits, thus influencing neuronal excitability and synaptic transmission. Persistent neuroimmune signaling may consequently facilitate a positive feedback cycle in which neurodegenerative injury promotes glial reactivity, glia-derived signals alter neuronal function, and altered neuronal activity further sustains neuroimmune responses. Recent mechanistic literature on chronic pain similarly places NLRP3-dependent signaling within neuronal, glial, and immune networks that can promote peripheral and central sensitization. Nevertheless, this model should be regarded as an interpretive framework rather than as evidence that all inflammatory pathways identified in neurodegenerative diseases contribute equally to pain.

Importantly, this review’s findings argue against equating neuroinflammation with pain. Neuroinflammatory processes are widespread features of neurodegenerative disorders, whereas pain phenotypes vary substantially across diseases, disease stages, anatomical regions, and experimental models. Furthermore, the included studies did not consistently demonstrate that more pronounced inflammatory or glial responses were associated with greater nociceptive sensitivity. This distinction is critical because glial responses may be detrimental, compensatory, protective, or functionally neutral depending on their cellular state and temporal context.

Neuroinflammation is therefore best interpreted as a shared but non-universal amplifier of nociceptive dysfunction rather than as a sufficient explanation for neurodegeneration-associated pain. Convergence among IL-6/IL-6R, HMGB1, NF-κB, NLRP3, and inflammatory cytokine signaling supports a substantial neuroimmune component, but heterogeneity across the included studies indicates these mechanisms operate within broader neuroglial networks. This interpretation provides a basis for extending the discussion beyond classical inflammation to neuron–glia communication, synaptic plasticity, ion-channel regulation, and descending modulatory systems.

### 4.3. Neuroglial and Synaptic Regulation

Although neuroinflammatory signaling emerged as a recurrent component of altered nociception, the included studies indicate that glial involvement extends beyond cytokine release and classical inflammatory pathways. Several mechanisms converge on regulating neuronal excitability, synaptic transmission, and descending pain control, including CX3CL1–CX3CR1, BDNF–TrkB–ERK, S1PR1-dependent signaling, serotonergic modulation, and α2-adrenergic receptor–Cav2.2 signaling. Collectively, these findings support a broader framework in which microglia and astrocytes participate in dynamic neuroglial networks that modify nociceptive processing at multiple levels of the central nervous system.

The CX3CL1–CX3CR1 axis represents one of the clearest examples of neuron–microglia communication identified in the included EAE studies. Zhu et al. [9] reported increased expression of fractalkine (CX3CL1) and its receptor, CX3CR1, in the dorsal root ganglia and spinal cord of animals with EAE, associated with neuropathic pain-related changes. This pathway is particularly relevant because CX3CL1 is expressed predominantly by neurons, whereas CX3CR1 is strongly associated with microglia in the central nervous system, providing a signaling interface through which neuronal alterations may influence microglial responses. Thus, rather than acting only as downstream inflammatory effector cells, microglia may actively detect changes in neuronal state and contribute to the remodeling of nociceptive circuits.

A complementary mechanism was identified by Khan et al. [17], who demonstrated that mechanical allodynia in an optimized relapsing-remitting EAE model was accompanied by increased BDNF–TrkB–ERK signaling in the dorsal gray matter of the spinal cord. Treatment with alpha-lipoic acid reduced allodynia and attenuated this signaling pathway. These findings matter because BDNF-dependent signaling may provide an interface between neuroimmune responses and activity-dependent changes in nociceptive neurons. Microglia-derived BDNF has been implicated in altered neuronal chloride homeostasis and inhibitory signaling in models of neuropathic pain, illustrating how glial responses may influence pain processing through synaptic and electrophysiological mechanisms rather than solely through inflammatory mediator release. However, the cellular origin and functional contribution of BDNF should be interpreted according to the experimental evidence available in each disease model rather than assumed to represent a universal microglial mechanism.

Findings related to S1PR1 further reinforce this concept. Doolen et al. [8] demonstrated that fingolimod reduced neuropathic pain-related behavior in an EAE model through an S1PR1-dependent mechanism associated with inhibition of central sensitization in the dorsal gray matter of the spinal cord. Importantly, the antinociceptive effect could be separated, at least in part, from changes in neurological disability, suggesting that modulation of nociceptive circuits was not merely secondary to improved motor function. This distinction is particularly relevant in neurodegenerative models, in which motor impairment may substantially confound behavioral pain assessment. S1PR1 signaling therefore represents a pathway operating at the intersection of immune regulation, glial function, and neuronal sensitization.

Evidence from Parkinsonian models extends this neuroglial framework to descending pain modulation. Campos et al. [22] demonstrated that motor cortex stimulation reversed hyperalgesia in a rat model of PD, increased serotonergic activity in raphe-related pathways, and was accompanied by inhibition of spinal astroglia responses. These observations are particularly informative because they connect supraspinal neuromodulation with descending monoaminergic systems and glial changes in the spinal cord. The findings suggest that astroglia responses in neurodegeneration-associated pain may not be driven exclusively by local inflammatory events but may also be dynamically regulated by the activity of descending neuronal networks.

A more recent PD study further extended this interaction to noradrenergic control of spinal nociceptive transmission. Cui et al. [29] implicated α2-adrenergic receptor signaling and Cav2.2-mediated neurotransmission within a spinal neuroglial network associated with nociception in Parkinson’s disease. Although this study should be interpreted cautiously because complete methodological information was not available for the present risk-of-bias assessment, its findings support the broader concept that monoaminergic modulation and voltage-gated calcium-channel activity may interact with glial responses to regulate nociceptive transmission. This mechanism is particularly relevant in PD, in which degeneration of monoaminergic systems extends beyond the nigrostriatal dopaminergic pathway and may affect endogenous pain-control networks.

Collectively, these studies indicate that glial contributions to pain in neurodegenerative diseases should not be conceptualized exclusively as an inflammatory cascade. Instead, the evidence points to reciprocal interactions among neurons, microglia, astrocytes, neurotransmitter systems, and synaptic signaling pathways. Within this framework, inflammatory mediators represent one component of a broader regulatory network in which glial cells may modify synaptic efficacy, neuronal excitability, ion-channel function, and descending modulatory control.

This interpretation is consistent with contemporary concepts of central sensitization, in which persistent pain is understood as a consequence of altered neuronal responsiveness maintained through interactions between neuronal and non-neuronal components of the central nervous system. Importantly, however, the evidence synthesized here suggests that the specific configuration of these interactions differs among neurodegenerative conditions. In EAE, spinal CX3CL1–CX3CR1, BDNF–TrkB–ERK, and S1PR1-related mechanisms were prominent, whereas Parkinsonian models highlighted interactions between spinal glia and descending serotonergic or noradrenergic systems. The available evidence therefore supports multiple disease-dependent routes leading to altered nociceptive processing rather than a single conserved glial pathway.

This broader neuroglial perspective also has therapeutic implications. Interventions that modified pain-related behavior in the included studies frequently affected pathways located at the interface between glial function and neuronal signaling rather than merely suppressing inflammation. Targeting specific neuron–glia signaling nodes might therefore offer greater mechanistic precision than broadly inhibiting glial reactivity. However, because most evidence remains preclinical and concentrated in EAE and Parkinsonian models, it remains unclear whether these mechanisms translate into clinically relevant therapeutic strategies.

Overall, the evidence reviewed in this section supports a transition from an inflammation-centered to a neuroglial network model of nociceptive dysfunction. In the latter, microglia and astrocytes interact with neuronal signaling systems at spinal and supraspinal levels, and their contribution to pain depends on the molecular pathway and neural circuit involved. This framework also provides a basis for understanding why astrocytes may exert functionally relevant effects independently of, concurrently with, or subsequent to microglial responses.

### 4.4. Context-Dependent Glial Regulation of Nociception

A central finding emerging from this systematic review is that the relationship between glial responses and nociception cannot be adequately explained by a unidirectional model in which greater microglial or astroglia reactivity necessarily leads to increased pain. Instead, the available evidence supports a model of context-dependent glial regulation in which the functional consequences of microglial and astroglia responses vary according to the underlying neurodegenerative disease, anatomical region, disease stage and severity, cellular state, and, when investigated, biological sex. This heterogeneity is not merely a limitation of the available literature. Rather, it provides important biological insight into the dynamic nature of neuroglial regulation of pain processing.

Temporal variation is particularly evident in EAE. Duffy et al. [2] demonstrated that pain-related behaviors and peripheral and central neuroinflammatory changes evolve throughout disease progression, indicating that nociceptive alterations cannot be interpreted independently of the temporal course of neuroinflammation. Similarly, Segal et al. [20] showed that chronic mechanical hypersensitivity in EAE was influenced by disease severity and neuroinflammatory status. Collectively, these findings suggest that the relationship among neuroinflammation, glial responses, and pain is dynamic rather than fixed. A glial response observed during disease induction may therefore have different functional implications from a phenotypically similar response detected during established or chronic disease.

This temporal dimension is further supported by the astrocytic findings discussed above. Ding et al. [11] identified an astrocyte-dependent component of early EAE-associated neuropathic pain at a stage in which prominent microglial involvement was not evident. When considered alongside studies examining later disease stages, this observation suggests that the relative contributions of different glial populations may change during disease progression. Consequently, cross-sectional measurements of Iba1, GFAP, cytokines, or other markers at a single time point may only partially represent the neuroglial processes associated with nociception.

Anatomical context represents another important concept. The included studies identified pain-associated glial alterations at multiple levels of the nociceptive system, including the dorsal root ganglia, spinal dorsal horn, striatum, amygdala, and other supraspinal structures. These regional differences matter because glial cells interact with distinct neuronal populations and circuits depending on their anatomical location. For example, spinal glial responses may directly influence sensory transmission and central sensitization, whereas glial changes in the amygdala may contribute to the affective and integrative dimensions of pain. Similar molecular signals therefore cannot be assumed to have identical functional consequences across different regions of the nervous system.

The findings of Dworsky-Fried et al. [24] are particularly informative. Their EAE study identified neuroinflammatory alterations in the central amygdala associated with pain hypersensitivity and altered morphine analgesia, showing that neuroimmune mechanisms relevant to pain extend beyond the spinal cord. Importantly, the study also identified sex-dependent features of the neuroimmune and behavioral responses. Although sex was not systematically examined across the entire evidence base, these findings highlight biological sex as a potentially important modifier of glial–nociceptive interactions. The limited incorporation of sex as an experimental variable in the included studies therefore represents both a methodological limitation and an important direction for future research.

Regional heterogeneity was also evident in the PD models. Zhang et al. [26] reported changes in striatal microglial and astrocytic responses associated with bradykinesia and allodynia during the early stages of Parkinsonian pathology. When considered alongside studies identifying microglial or astrocytic alterations in the spinal cord [3,22,27,29] and microglial inflammasome signaling in the amygdala [30], these findings suggest that pain-related neuroglial dysfunction in PD spans multiple levels of the nervous system rather than being confined to a single nociceptive structure. This spatial organization may be particularly relevant in PD, in which degeneration of dopaminergic and non-dopaminergic systems may simultaneously alter sensory processing, affective integration, and descending pain modulation.

Perhaps the strongest evidence against a simple relationship between greater glial reactivity and increased pain comes from the AD literature. Sideris-Lampretsas et al. [10] investigated inflammatory nociception in wild-type mice and TASTPM mice modeling Alzheimer’s disease. In wild-type animals, galectin-3 induced inflammatory nociception through spinal microglial mechanisms involving innate immune and purinergic signaling. However, this response was altered in the AD model, in which disease-associated microglial changes were accompanied by attenuated rather than increased inflammatory allodynia. Thus, a neurodegeneration-associated microglial phenotype did not simply increase susceptibility to pain.

This finding is conceptually important because it indicates that microglial reactivity is not intrinsically pronociceptive. The functional consequences of a microglial state depend on the molecular and cellular environment in which it develops. Chronic neurodegenerative pathology may alter receptor expression, intracellular signaling, responsiveness to damage-associated signals, or communication between microglia and nociceptive neurons. Consequently, microglia chronically exposed to amyloid-related pathology may respond differently to an acute nociceptive challenge than microglia within a neurologically intact nervous system. The findings of Sideris-Lampretsas et al. [10] therefore provide direct experimental support for interpreting glial state, rather than merely the magnitude of glial reactivity, as a critical determinant of nociceptive outcomes.

Collectively, the studies included in this systematic review support a model in which disease context modifies the functional meaning of glial reactivity. EAE provides evidence of temporal changes associated with disease progression and severity [2,20], as well as early astrocytic contributions that do not require prominent concomitant microglial changes [11]. Parkinsonian models demonstrate spatially distributed glial mechanisms involving spinal, striatal, and supraspinal circuits [3,22,26,27,29,30]. Meanwhile, evidence from AD shows that a disease-associated microglial state may attenuate rather than amplify a nociceptive response [10]. Taken together, these observations argue against treating glial activation as a uniform mechanistic endpoint.

We therefore propose that glial regulation of nociception in neurodegenerative diseases is best conceptualized as a dynamic and multidimensional process. Within this framework, neurodegenerative pathology modifies microglial and astrocytic states; these states interact with region-specific neuronal circuits and molecular signaling pathways; and disease stage, severity, sex, and experimental conditions further influence their functional consequences. The resulting phenotype may range from increased nociceptive sensitivity to altered analgesic responsiveness or, under particular conditions, attenuation of nociceptive responses.

Importantly, this model does not imply that each contextual factor identified in the included studies has been shown to exert an independent causal effect. Rather, it represents an integrative framework derived from the heterogeneity observed across the evidence base. Disease type, anatomical compartment, temporal stage, and sex should therefore be regarded as potential modifiers of glial–nociceptive interactions, with evidence strength varying across these dimensions.

The central conceptual implication of this review is therefore that greater glial reactivity should not automatically be interpreted as greater pain. The more relevant questions concern which microglial or astrocytic state is present, where and when it occurs, which molecular pathway is engaged, and how that state interacts with the neuronal circuit responsible for nociceptive processing. This context-dependent framework provides a more biologically plausible interpretation of apparently heterogeneous findings across neurodegenerative disease models and forms the basis of the integrative model proposed in this review.

### 4.5. Methodological Considerations, Limitations, and Future Research Directions

The findings should be interpreted in light of several methodological limitations. Risk-of-bias assessment was independently performed by two investigators using SYRCLE, and a lack of methodological reporting was classified as unclear rather than low risk. Particular concerns included incomplete reporting of sequence generation, allocation concealment, random housing, and blinding; possible attrition related to EAE severity/humane endpoints; and selection of successfully lesioned animals in PD models. Female animals predominated in EAE studies, relatively few studies included both sexes, and age was inconsistently reported; most experiments used young or young-adult rodents despite aging being central to several neurodegenerative diseases. Sex-dependent microglial mechanisms described in other pain paradigms therefore cannot be assumed to generalize to this evidence base, but they underscore the need to treat sex as a biological variable.

Cell identity also limits interpretation. IBA1 and CD11b are not unequivocally specific for resident microglia in inflammatory models such as EAE, and increased GFAP does not by itself define a functional astrocyte state. Future studies should incorporate cell-specific markers or lineage/manipulation strategies, matched behavioral and glial time points, and explicit reporting of whether interventions alter lesion severity, EAE clinical score, or motor performance. When disease-modifying and nociceptive effects cannot be separated, direct antinociception should remain an unresolved explanation rather than an assumption.

Explicit adherence to ARRIVE guidelines was reported only in a subset of the included studies. Absence of an ARRIVE statement does not establish non-compliance; nevertheless, prospective use of ARRIVE 2.0 [36] would improve reporting of randomization, blinding, sample size justification, exclusions, attrition, and experimental unit definitions. The registered PROSPERO protocol also planned broader database coverage than was originally implemented. The addition of Scopus and Web of Science during revision improved coverage, but Embase and Cochrane Library were not searched; this protocol deviation and the inability to reconstruct a valid historical Cohen’s kappa are acknowledged as limitations.

## 5. Conclusions

The available preclinical evidence supports context-dependent relationships between glial responses and altered nociceptive processing in neurodegenerative disease models, but it does not support a uniform pronociceptive role for microglia or astrocytes. The evidence is dominated by MS/EAE and PD models, while AD and HD conclusions rely on very few studies. Associations involving inflammatory, neuroimmune, synaptic, and astrocyte-related pathways should therefore be interpreted according to disease model, anatomical region, disease stage, marker specificity, sex, age, and whether the experimental manipulation directly targeted the relevant cell population. Findings supported by single studies remain hypothesis-generating and require replication before clinical translation.

Collectively, these findings suggest that microglia and astrocytes should be regarded as dynamic components of neuroglial networks rather than as uniform markers or drivers of neuroinflammation. The limited evidence available for Alzheimer’s and Huntington’s diseases, the absence of eligible studies on amyotrophic lateral sclerosis, and the methodological heterogeneity of the current literature highlight the need for a broader and more rigorously characterized range of experimental models. Glial regulation of nociception in neurodegenerative diseases is therefore best understood as a dynamic, context-dependent neuroglial process rather than a uniform consequence of generalized glial activation.

## Figures and Tables

**Table 1:** The characteristics of the studies included in the systematic review.

Study	Disease/Model	Animals (Species/Strain/Sex/Age or Weight)	Glial Component	Pain-Related Outcome	Intervention/Main Pathway	Main Finding
Dutra et al. [12]	MS/EAE	C57BL/6 mice, female	Astrocytes	Tactile and thermal hypersensitivity	Kinin B1/B2 receptors	Genetic/pharmacological modulation of B1/B2 receptors reduced hypersensitivity and inflammatory glial responses; B1R was associated with astrocytes.
Zhu et al. [9]	MS/MBP-EAE	Lewis rats, female	Microglia + astrocytic/neuronal signaling	Thermal sensory abnormalities	CX3CL1–CX3CR1	CX3CL1/CX3CR1 increased in DRG and spinal cord in association with sensory abnormalities; microglia were an important spinal CX3CR1-expressing population.
Khan et al. [17]	MS/EAE	C57BL/6 mice, female	Microglia	Mechanical allodynia	α-Lipoic acid; BDNF–TrkB–ERK	Treatment reduced allodynia, microglial reactivity, and BDNF–TrkB–ERK signaling.
Duffy et al. [2]	MS/EAE	C57BL/6J mice	Microglia + astrocytes	Hind paw/facial mechanical allodynia; spontaneous pain	Disease-stage neuroinflammation	Pain behaviors varied across disease progression and were associated with region- and stage-dependent glial changes.
Doolen et al. [8]	MS/MOG-EAE	C57BL/6 mice, female, 8–12 wk	Microglia + astrocytes	Mechanical and cold hypersensitivity	Fingolimod/S1PR1	Fingolimod reduced hypersensitivity before substantial motor recovery and attenuated indices of dorsal-horn central sensitization and gliosis.
Serizawa et al. [18]	MS/MOG-EAE	C57BL/6J mice, female	Microglia	Mechanical hypersensitivity	Anti-IL-6R antibody	IL-6R blockade reduced pain sensitivity and spinal microglial/inflammatory responses.
Jin et al. [19]	AD/rTg4510	Transgenic mice	Microglia	Neuropathic pain-related behavior	TMEM119-targeted DNMT1 modulation	Microglial reprogramming was associated with attenuation of pain-related behavior.
Segal et al. [20]	MS/MOG-EAE	C57BL/6 mice	Microglia/macrophages + astrocytes	Mechanical and cold hypersensitivity	Modification of EAE severity	Pain detectability and glial responses depended strongly on disease severity; severe motor impairment represented an important behavioral confounder.
Teixeira et al. [21]	MS/MOG-EAE	C57BL/6 mice, female	Microglia/macrophages	Mechanical hypernociception	Crotoxin	Crotoxin produced antinociceptive and immunomodulatory effects accompanied by reduced activated microglial/macrophage responses.
Campos et al. [22]	PD/6-OHDA	Rats; sex/age as reported in primary study	Astrocytes + microglia	Mechanical hypernociception (specific assay as reported)	Motor cortex stimulation/descending serotonin	Motor cortex stimulation reversed hypernociception, increased raphe serotonergic activity, and reduced spinal astrocytic reactivity, whereas microglial changes were less affected.
Liu et al. [23]	HD/transgenic model	Transgenic mice	Microglia + astrocytes	Thermal nociception; morphine analgesia	Spinal neuroinflammation	Premanifest HD was associated with altered morphine analgesia and a modified spinal glial/inflammatory response.
Sato et al. [3]	PD/unilateral 6-OHDA	Mice	Microglia	Mechanical hypersensitivity	HMGB1 neutralization	Anti-HMGB1 treatment reduced mechanical hypersensitivity and spinal microglial activation without requiring recovery of the dopaminergic lesion.
Ding et al. [11]	MS/EAE	C57BL/6 mice, female	Astrocytes	Early mechanical hypersensitivity	LRRC8A–STAT3	Astrocytic reactivity preceded prominent microglial involvement; astrocyte inhibition and LRRC8A blockade attenuated early pain.
Dworsky-Fried et al. [24]	MS/EAE	C57BL/6 mice, male and female	Microglia	Pain hypersensitivity; morphine analgesia	Central amygdala neuroinflammation	Amygdala microglial alterations were associated with pain hypersensitivity and modified opioid analgesia, with sex-dependent effects.
Dalenogare et al. [25]	MS/relapsing-remitting and progressive EAE	Mice	Microglia + astrocytes	Mechanical and cold allodynia	TRPA1 deletion	TRPA1 deletion attenuated pain-like behaviors and reduced neuroinflammatory microglial/astrocytic responses.
Sideris-Lampretsas et al. [10]	AD/TASTPM	Transgenic mice, male and female	Microglia	Inflammatory mechanical allodynia	Galectin-3–TLR4–P2Y12/p38	AD pathology altered the spinal microglial response and was associated with reduced inflammatory allodynia rather than a simple increase in pain.
Zhang et al. [26]	PD/unilateral 6-OHDA	Mice	Microglia + astrocytes	Mechanical allodynia	Minocycline/striatal glial states	6-OHDA produced mechanical allodynia and reactive glial programs in the dorsal striatum; minocycline attenuated both glial alterations and allodynia.
Godínez-Chaparro et al. [27]	PD/unilateral 6-OHDA	Wistar rats, male	Microglia + astrocytes	Mechanical allodynia/hyperalgesia	Pramipexole/astrocytic NF-κB	Pramipexole attenuated mechanical hypersensitivity and spinal glial reactivity, including astrocyte-associated NF-κB/p65 signaling.
Magni et al. [28]	MS/EAE	Dark Agouti rats, male, ~200 g	Microglia + astrocytes	Trigeminal pain/allodynia	Anthocyanin-enriched purple corn extract	Preventive treatment reduced trigeminal pain and attenuated neuroinflammatory microgliosis and astrogliosis.
Cui et al. [29]	PD/6-OHDA + DSP-4	Sprague Dawley rats, male, 6–8 wk, 180–220 g	GFAP+ astrocytes; IBA1+ microglia/macrophage-associated cells	Mechanical and thermal hypersensitivity	α2-adrenergic receptor–Cav2.2	Noradrenergic dysfunction was associated with spinal gliosis, Cav2.2-related neuronal excitability and hypersensitivity; pharmacological modulation reduced these responses.
Wang et al. [30]	PD/MPTP	C57BL/6 mice, male	Microglia	Mechanical and thermal nociception	Electroacupuncture/NLRP3–ASC–caspase-1	Electroacupuncture increased pain thresholds and attenuated microglial inflammasome signaling and pro-inflammatory cytokines.
Schmitz et al. [31]	MS/EAE	CXCR3−/− and plt/plt mice, C57BL/6 background, male and female, 12–16 wk	CD11b/IBA1 microglia/macrophage-associated cells; inflammatory infiltrate	Mechanical, cold and thermal hyperalgesia	CCL21/CXCR3	CXCR3 deficiency reduced EAE-evoked hyperalgesia and inflammatory infiltration; CD11b/IBA1 findings were interpreted as myeloid/microglia-associated rather than resident-microglia-specific.
Giardini et al. [32]	MS/MOG-EAE	C57BL/6J mice, female	Spinal glial reactivity; inflammatory infiltrate	Mechanical hyperalgesia	Crotalphine/CB2-related signaling	Crotalphine partially reversed mechanical hyperalgesia and reduced spinal inflammatory/glial responses and EAE clinical severity.
Gao et al. [33]	PD/6-OHDA	Rats	GFAP+ astrocytic reactivity in prefrontal cortex, anterior cingulate gyrus and striatum	Heat sensitivity threshold; pressure withdrawal threshold	Norepinephrine/α2-adrenergic receptor	Noradrenergic manipulation altered nociceptive thresholds together with GFAP-positive cell responses; systemic/disease-related effects remain potential contributors.
Evangelista et al. [34]	MS/MOG-EAE	ALDH2*2 knock-in and C57BL/6 WT mice, female, 18–22 g	GFAP and IBA1 spinal glial reactivity	Mechanical hypernociception	ALDH2/Alda-1	ALDH2 deficiency aggravated EAE hypernociception and glial reactivity; Alda-1 improved behavioral and neuroinflammatory outcomes.
Morozzi et al. [35]	MS/MOG-EAE	C57BL/6 mice, female, 6–10 wk, 20–25 g	Iba1-associated spinal microglial/myeloid reactivity	Mechanical and thermal hypernociception	A2A receptor antagonists P400/P625	P400/P625 attenuated nociceptive and motor symptoms; P400 reduced Iba1 immunostaining and both compounds reduced neuroinflammation/demyelination.

Abbreviations: MS, multiple sclerosis; EAE, experimental autoimmune encephalomyelitis; PD, Parkinson’s disease; AD, Alzheimer’s disease; HD, Huntington’s disease; 6-OHDA, 6-hydroxydopamine; MPTP, 1-methyl-4-phenyl-1,2,3,6-tetrahydropyridine; DRG, dorsal root ganglion; BDNF, brain-derived neurotrophic factor; S1PR1, sphingosine-1-phosphate receptor 1; HMGB1, high-mobility group box 1; TRPA1, transient receptor potential ankyrin 1; TLR4, Toll-like receptor 4; DSP-4, N-(2-chloroethyl)-N-ethyl-2-bromobenzylamine; IBA1, ionized calcium-binding adapter molecule 1; GFAP, glial fibrillary acidic protein; ALDH2, aldehyde dehydrogenase 2; CB2, cannabinoid receptor type 2; P2Y12, purinergic receptor P2Y12.

**Table 2:** Functional synthesis of glial evidence associated with pain and nociceptive alterations across neurodegenerative disease models.

Functional Module	Molecular/Cellular Mechanisms Identified	Glial Component	Disease Models	Representative Studies	Main Finding Across Studies
**I. Neuroinflammatory amplification**	NLRP3–ASC–caspase-1; IL-1β; IL-6/IL-6R; TNF-α; HMGB1; NF-κB/p65	Predominantly microglia, with astrocytic participation	PD; MS/EAE	Serizawa et al. [18]; Sato et al. [3]; Godínez-Chaparro et al. [27]; Wang et al. [30]	Enhanced inflammatory signaling was generally associated with nociceptive hypersensitivity and reactive glial responses. Experimental attenuation of these pathways was accompanied by reduced pain-related behavior and/or glial inflammatory responses.
**II. Neuroglial and synaptic signaling**	CX3CL1–CX3CR1; BDNF–TrkB–ERK; S1PR1; descending serotonergic signaling; α2-adrenergic receptor–Cav2.2	Microglia and astrocytes interacting with neuronal pathways	MS/EAE; PD	Zhu et al. [9]; Khan et al. [17]; Doolen et al. [8]; Campos et al. [22]; Cui et al. [29]	Glial alterations were linked to neuronal excitability, descending pain modulation, and central sensitization, indicating that nociceptive dysfunction cannot be explained by inflammatory signaling alone.
**III. Astrocyte-dependent and multimodal glial regulation**	LRRC8A–STAT3; glutamate-related signaling; kinin B1/B2 receptors; TRPA1	Astrocytes alone or combined astrocytic–microglial responses	Predominantly MS/EAE	Dutra et al. [12]; Ding et al. [11]; Dalenogare et al. [25]	Astrocytes showed functionally relevant contributions to nociceptive hypersensitivity, including early disease stages in which prominent microglial involvement was not evident. Other pathways involved coordinated astrocytic and microglial responses.
**IV. Context-dependent glial regulation**	Galectin-3–TLR4–P2Y12/p38; disease-associated microglial states; temporal evolution of gliosis; regional glial responses; sex-related differences	Microglia and astrocytes	AD; MS/EAE; HD; PD	Duffy et al. [2]; Segal et al. [20]; Liu et al. [23]; Dworsky-Fried et al. [24]; Sideris-Lampretsas et al. [10]; Zhang et al. [26]	The relationship between glial reactivity and pain varies according to disease, anatomical region, disease stage/severity, and experimental context. Increased glial reactivity did not invariably correspond to greater nociceptive sensitivity.

Abbreviations: AD, Alzheimer’s disease; BDNF, brain-derived neurotrophic factor; EAE, experimental autoimmune encephalomyelitis; HD, Huntington’s disease; HMGB1, high-mobility group box 1; IL, interleukin; MS, multiple sclerosis; NF-κB, nuclear factor kappa B; PD, Parkinson’s disease; S1PR1, sphingosine-1-phosphate receptor 1; TNF-α, tumor necrosis factor alpha; TRPA1, transient receptor potential ankyrin 1. Bold text indicates the four functional modules used to organize the qualitative synthesis.

## Data Availability

Review-generated data supporting this systematic review, including database-specific search strategies and retrieval counts, study-level extracted data, and detailed SYRCLE risk-of-bias judgments, are provided in the Supplementary Materials. Additional screening documentation is available from the corresponding author upon reasonable request.

## Supplementary Materials

The following supporting information can be downloaded at: https://www.mdpi.com/article/10.3390/brainsci16101093/s1, Table S1: Database-specific search strategies, search dates, coverage, and retrieval counts; Table S2: Study-level characteristics, glial/cell-identity evidence, anatomical context, nociceptive phenotype, and principal pathway for the 26 included studies; Table S3: Study-level SYRCLE risk-of-bias judgments for the 26 included studies; Table S4: PRISMA 2020 Checklist.
