# Supplementary material for: Glial Contributions to Pain in Neurodegenerative Disease Models: A Systematic Review of Microglial and Astrocytic Evidence
Source: Brain Sci. 2026 Oct 10;16(10):1093. doi: 10.3390/brainsci16101093 (PMC13702446; doi:10.3390/brainsci16101093)
Supplement: Supplementary file 1 [file brainsci-16-01093-s001.zip › brainsci-4576642-supplementary.pdf]

Supplementary Table S1. Database-specific search strategies, search dates, coverage, and retrieval counts.

Source / platform	Search date	Coverage	Exact search strategy / reporting note	Limits / field	Records retrieved
PubMed/MEDLINE (NCBI PubMed)	24 Aug 2026	1 Jan 2000–24 Aug 2026	The preserved PubMed export documents the original retrieval (n=437), but the exact historical query string was not retained. It is therefore not retrospectively reconstructed or presented as the original query.	No language restriction at electronic retrieval; English/Spanish eligibility applied during study selection.	437
Scopus	25 Sep 2026	2000–2026	TITLE-ABS-KEY((microglia* OR "microglial activation" OR "microglial dysfunction" OR "microglial reactivity" OR "microglial state" OR astrocyt* OR astroglia* OR astrogliosis OR "astrocyte activation" OR "astrocyte dysfunction" OR "reactive astrocyte" OR "reactive astrocytes") AND (pain OR nocicept* OR hyperalgesi* OR allodyni* OR hypersensitiv* OR hypernocicept* OR analges* OR "pain perception" OR "pain modulation" OR "nociceptive processing" OR "nociceptive signaling" OR "central sensitization") AND ("neurodegenerative disease" OR "neurodegenerative diseases" OR neurodegenerat* OR "Parkinson disease" OR "Parkinson's disease" OR parkinson* OR "Alzheimer disease" OR "Alzheimer's disease" OR alzheimer* OR "multiple sclerosis" OR "experimental autoimmune encephalomyelitis" OR EAE OR "amyotrophic lateral sclerosis" OR ALS OR "Huntington disease" OR "Huntington's disease" OR huntington*)) AND PUBYEAR > 1999 AND PUBYEAR < 2027	TITLE-ABS-KEY; PUBYEAR >1999 and <2027	1,381
Web of Science Core Collection	25 Sep 2026	2000–2026	(microglia* OR "microglial activation" OR "microglial dysfunction" OR "microglial reactivity" OR "microglial state" OR astrocyt* OR astroglia* OR astrogliosis OR "astrocyte activation" OR "astrocyte dysfunction" OR "reactive astrocyte" OR "reactive astrocytes") AND (pain OR nocicept* OR hyperalgesi* OR allodyni* OR hypersensitiv* OR hypernocicept* OR analges* OR "pain perception" OR "pain modulation" OR "nociceptive processing" OR "nociceptive signaling" OR "central sensitization") AND ("neurodegenerative disease" OR "neurodegenerative diseases" OR neurodegenerat* OR "Parkinson disease" OR "Parkinson's disease" OR parkinson* OR "Alzheimer disease" OR "Alzheimer's disease" OR alzheimer* OR "multiple sclerosis" OR "experimental autoimmune encephalomyelitis" OR EAE OR "amyotrophic lateral sclerosis" OR ALS OR "Huntington disease" OR "Huntington's disease" OR huntington*)	Topic field; Timespan 2000–2026	430

Deduplication: 437 + 1,381 + 430 = 2,248 raw records. Hierarchical deduplication by DOI → PMID → normalized title removed 681 duplicates, leaving 1,567 unique records for screening. The supplementary Scopus and Web of Science searches were conducted during major revision; Embase and Cochrane Library remained protocol-planned but unexecuted sources.

Supplementary Table S2. Study-level characteristics, glial/cell-identity evidence, anatomical context, nociceptive phenotype, and principal pathway for the 26 included studies.

Study	Disease/model	Animals / sex / age-weight	Glial markers / evidence	Cell-identity caveat	Region	Pain/nociceptive phenotype	Intervention / pathway	Principal finding
Dutra et al. [12]	MS / EAE	C57BL/6; B1R−/−; B2R−/− mice; sex: Female; age/weight: 6–10 wk	GFAP; Iba1	Iba1 does not separate infiltrating macrophages	Spinal cord	Tactile and thermal hypersensitivity	Kinin B1/B2 receptors	Genetic/pharmacological modulation of B1/B2 receptors reduced hypersensitivity and inflammatory glial responses; B1R was associated with astrocytes.
Zhu et al. [9]	MS / MBP-EAE	Lewis rats; sex: Female; age/weight: 6–8 wk	GFAP; CX3CL1/CX3CR1 localization	DRG identity not resolved as CNS microglia/astrocytes	DRG + spinal cord	Thermal sensory abnormalities	CX3CL1–CX3CR1	CX3CL1/CX3CR1 increased in DRG and spinal cord in association with sensory abnormalities; microglia were an important spinal CX3CR1-expressing population.
Khan et al. [17]	MS / EAE	C57BL/6 mice; sex: Female; age/weight: 4–6 wk	CD11b; Iba1	Myeloid/microglial marker; infiltration possible in EAE	Lumbar dorsal horn	Mechanical allodynia	α-Lipoic acid; BDNF–TrkB–ERK	Treatment reduced allodynia, microglial reactivity, and BDNF–TrkB–ERK signaling.
Duffy et al. [2]	MS / EAE	C57BL/6J mice; sex: Female; age/weight: 10–12 wk	Iba1; GFAP; immune markers	Iba1 alone not microglia-specific in EAE	Spinal cord + trigeminal pathway	Hind paw/facial mechanical allodynia; spontaneous pain	Disease-stage neuroinflammation	Pain behaviors varied across disease progression and were associated with region- and stage-dependent glial changes.
Doolen et al. [8]	MS / MOG-EAE	C57BL/6 mice; sex: Female; age/weight: NR in extracted passage	GFAP; Iba1	Iba1 limitation in EAE	Lumbar dorsal horn	Mechanical and cold hypersensitivity	Fingolimod / S1PR1	Fingolimod reduced hypersensitivity before substantial motor recovery and attenuated indices of dorsal-horn central sensitization and gliosis.
Serizawa et al. [26]	MS / MOG-EAE	C57BL/6J mice; sex: Female; age/weight: 7 wk	Iba1; CD45lowCD11b+ flow cytometry	Flow cytometry improves resident microglia discrimination	Spinal cord	Mechanical hypersensitivity	Anti-IL-6R antibody	IL-6R blockade reduced pain sensitivity and spinal microglial/inflammatory responses.
Jin et al. [18]	AD / rTg4510	rTg4510 transgenic mice; sex: NR in available text; age/weight: 5 mo at testing	TMEM119 promoter; microglial phenotype markers	TMEM119-targeted manipulation supports microglial specificity	CA3	Neuropathic pain-related behavior	TMEM119-targeted DNMT1 modulation	Microglial reprogramming was associated with attenuation of pain-related behavior.
Segal et al. [19]	MS / MOG-EAE	C57BL/6J mice; sex: Female; age/weight: 7–10 wk	GFAP; CD11b; flow cytometry immune markers	Myeloid populations require marker combinations	Spinal cord	Mechanical and cold hypersensitivity	Modification of EAE severity	Pain detectability and glial responses depended strongly on disease severity; severe motor impairment represented an important behavioral confounder.
Teixeira et al. [30]	MS / MOG-EAE	C57BL/6 mice; sex: Female; age/weight: 8–12 wk	CD11b/CD45; immune phenotyping	CD45 intensity helps distinguish microglia/macrophages	CNS / spinal tissue	Mechanical hypernociception	Crotoxin	Crotoxin produced antinociceptive and immunomodulatory effects accompanied by reduced activated microglial/macrophage responses.
Campos et al. [28]	PD / 6-OHDA	Wistar rats; sex: Male; age/weight: 200–250 g	GFAP; Iba1	Iba1 used as microglial marker	Spinal dorsal horn	Mechanical hypernociception (specific assay as reported)	Motor cortex stimulation / descending serotonin	Motor cortex stimulation reversed hypernociception, increased raphe serotonergic activity, and reduced spinal astrocytic reactivity, whereas microglial changes were less affected.
Liu et al. [20]	HD / transgenic model	Transgenic HD mice; sex: Mixed sex; age/weight: Young 2–4 mo	GFAP; Iba1	Iba1 limitation outside infiltrative EAE less severe but not absolute	Lumbar spinal cord	Thermal nociception; morphine analgesia	Spinal neuroinflammation	Premanifest HD was associated with altered morphine analgesia and a modified spinal glial/inflammatory response.
Sato et al. [3]	PD / unilateral 6-OHDA	ddY mice; sex: Male; age/weight: 4 wk at purchase	Iba1	Iba1-based microglial reactivity	Spinal cord	Mechanical hypersensitivity	HMGB1 neutralization	Anti-HMGB1 treatment reduced mechanical hypersensitivity and spinal microglial activation without requiring recovery of the dopaminergic lesion.
Ding et al. [11]	MS / EAE	C57BL/6 mice; sex: Female; age/weight: 6–8 wk, 18–20 g	GFAP; Iba1; astrocytic state markers including S100A10	Iba1 limitation in EAE	Spinal dorsal horn	Early mechanical hypersensitivity	LRRC8A–STAT3	Astrocytic reactivity preceded prominent microglial involvement; astrocyte inhibition and LRRC8A blockade attenuated early pain.
Dworsky-Fried et al. [29]	MS / EAE	C57BL/6 mice; sex: Male + female; age/weight: 10–12 wk	Iba1; CD4	Iba1 alone not specific in EAE; CD4 assesses infiltration	Central amygdala	Pain hypersensitivity; morphine analgesia	Central amygdala neuroinflammation	Amygdala microglial alterations were associated with pain hypersensitivity and modified opioid analgesia, with sex-dependent effects.
Dalenogare et al. [21]	MS / relapsing-remitting and progressive EAE	Trpa1+/+ / Trpa1−/− mice; sex: Female; age/weight: weight 15–30 g; age NR	Iba1; GFAP	Iba1 limitation in EAE	Spinal cord	Mechanical and cold allodynia	TRPA1 deletion	TRPA1 deletion attenuated pain-like behaviors and reduced neuroinflammatory microglial/astrocytic responses.
Sideris-Lampretsas et al. [10]	AD / TASTPM	TASTPM mice; sex: Male + female; age/weight: 6 mo	IBA1; P2Y12; TLR4; p-p38	P2Y12/TLR4 phenotyping improves state resolution	Spinal dorsal horn	Inflammatory mechanical allodynia	Galectin-3–TLR4–P2Y12/p38	AD pathology altered the spinal microglial response and was associated with reduced inflammatory allodynia rather than a simple increase in pain.
Zhang et al. [22]	PD / unilateral 6-OHDA	C57BL/6 WT mice; sex: Male; age/weight: 5–7 mo	Iba1; Gfap + state-associated genes	Gene panels exceed single-marker reactivity but M1/M2/A1/A2 terminology remains simplified	Dorsal striatum	Mechanical allodynia	Minocycline / striatal glial states	6-OHDA produced mechanical allodynia and reactive glial programs in the dorsal striatum; minocycline attenuated both glial alterations and allodynia.

Study	Disease/model	Animals / sex / age-weight	Glial markers / evidence	Cell-identity caveat	Region	Pain/nociceptive phenotype	Intervention / pathway	Principal finding
Godínez-Chaparro et al. [27]	PD / unilateral 6-OHDA	Wistar rats; sex: Male; age/weight: 280–300 g; age NR	GFAP; Iba1; NF-κB/p65	Iba1/GFAP reactivity; astrocytic NF-κB localization	L4–L6 dorsal horn	Mechanical allodynia/hyperalgesia	Pramipexole / astrocytic NF-κB	Pramipexole attenuated mechanical hypersensitivity and spinal glial reactivity, including astrocyte-associated NF-κB/p65 signaling.
Magni et al. [23]	MS / EAE	Dark Agouti rats; sex: Male; age/weight: ~200 g; age NR	Iba1; GFAP	Iba1 limitation in EAE	Brainstem + cervical/lumbar spinal cord	Trigeminal pain/allodynia	Anthocyanin-enriched purple corn extract	Preventive treatment reduced trigeminal pain and attenuated neuroinflammatory microgliosis and astrogliosis.
Cui et al. [24]	PD / 6-OHDA + DSP-4	[FULL TEXT NOT RE-UPLOADED]; sex: NR; age/weight: NR	GFAP; IBA1	GFAP denotes astrocytic reactivity; IBA1 is interpreted as microglia/macrophage-associated immunoreactivity.	Spinal dorsal horn	Mechanical and thermal hypersensitivity	α2-adrenergic receptor–Cav2.2	Noradrenergic dysfunction was associated with spinal gliosis, Cav2.2-related neuronal excitability and hypersensitivity; pharmacological modulation reduced these responses.
Wang et al. [25]	PD / MPTP	C57BL/6 mice (per manuscript); sex: Male (per manuscript); age/weight: NR	[VERIFY]	Cannot independently verify	[VERIFY]	Mechanical and thermal nociception	Electroacupuncture / NLRP3–ASC–caspase-1	Electroacupuncture increased pain thresholds and attenuated microglial inflammasome signaling and pro-inflammatory cytokines.
Schmitz et al. [31]	MS / EAE	CXCR3−/− and plt/plt mice, C57BL/6 background, male and female, 12–16 wk	CD11b; IBA1; inflammatory infiltrate	CD11b/IBA1 cannot unequivocally distinguish resident microglia from infiltrating macrophages in EAE.	Spinal cord	Mechanical, cold and thermal hyperalgesia	CCL21 / CXCR3	CXCR3 deficiency reduced EAE-evoked hyperalgesia and inflammatory infiltration; CD11b/IBA1 findings were interpreted as myeloid/microglia-associated rather than resident-microglia-specific.
Giardini et al. [32]	MS / MOG-EAE	C57BL/6J mice, female	Spinal glial-cell activation; inflammatory infiltrate	Glial activation reported in an infiltrative EAE context; resident microglia specificity should not be inferred from nonspecific myeloid markers.	Spinal cord	Mechanical hyperalgesia	Crotalphine / CB2-related signaling	Crotalphine partially reversed mechanical hyperalgesia and reduced spinal inflammatory/glial responses and EAE clinical severity.
Gao et al. [33]	PD / 6-OHDA	Rats	GFAP-positive astrocytic reactivity	GFAP indicates astrocytic reactivity but does not define a functional astrocyte state.	Prefrontal cortex; anterior cingulate gyrus; striatum	Heat sensitivity threshold; pressure withdrawal threshold	Norepinephrine / α2-adrenergic receptor	Noradrenergic manipulation altered nociceptive thresholds together with GFAP-positive cell responses; systemic/disease-related effects remain potential contributors.
Evangelista et al. [34]	MS / MOG-EAE	ALDH2*2 knock-in and C57BL/6 WT mice, female, 18–22 g	GFAP; IBA1	GFAP indicates astrocytic reactivity; IBA1 in EAE is not resident-microglia-specific.	L4–L6 spinal dorsal horn	Mechanical hypernociception	ALDH2 / Alda-1	ALDH2 deficiency aggravated EAE hypernociception and glial reactivity; Alda-1 improved behavioral and neuroinflammatory outcomes.
Morozzi et al. [35]	MS / MOG-EAE	C57BL/6 mice, female, 6–10 wk, 20–25 g	Iba1-associated microglial/myeloid reactivity	Iba1 alone does not distinguish resident microglia from infiltrating myeloid cells in EAE.	Spinal cord	Mechanical and thermal hypernociception	A2A receptor antagonists P400/P625	P400/P625 attenuated nociceptive and motor symptoms; P400 reduced Iba1 immunostaining and both compounds reduced neuroinflammation/demyelination.

Supplementary Table S3. Study-level SYRCLE risk-of-bias judgments for the 26 included studies.

Study	Disease	Seq. gen.	Baseline	Alloc. conceal.	Random housing	Blinding caregivers / investigators	Random outcome assess.	Blinding outcome assessor	Incomplete data	Selective reporting	Other bias	Key evidence / interpretation
Dutra et al.	MS/EAE	U	U	U	U	U	U	U	U	U	U	Prespecified exclusion of mice that did not develop EAE; no explicit randomization or blinding identified in the supplied article. Predominantly unclear because core bias-control procedures were not reported.
Zhu et al.	MS/EAE	L	U	U	U	U	U	U	U	U	U	Rats were randomly assigned to naïve control, active control, and EAE groups. Random sequence generation supported; other domains insufficiently reported.
Khan et al.	MS/EAE	L	U	U	U	L	U	L	U	U	U	Animals were randomized in a blinded manner; dosing solutions were masked; behavioral testing and IHC quantification were blinded. One of the best-reported studies for randomization and blinding, but concealment/random housing/attrition remain unclear.
Duffy et al.	MS/EAE	U	U	U	U	U	U	L	U	U	U	Hindpaw, facial allodynia, grimace scoring, and image analyses were performed blind to experimental groups. Detection-bias control is well supported; randomization and concealment remain unclear.
Doolen et al.	MS/EAE	U	U	U	U	U	U	L	U	U	U	Behavioral measurements and tissue quantification were performed by investigators blinded to group/treatment identity. Detection-bias control supported; selection/performance domains remain unclear.
Serizawa et al.	MS/EAE	U	U	U	U	U	U	U	U	U	U	Spinal cord slices were randomly selected, but group randomization/blinding were not identified in the supplied article. Most domains remain unclear.
Jin et al.	AD	U	U	U	U	U	U	U	U	U	U	No explicit randomization/blinding wording was identified in the supplied full text search. Predominantly unclear reporting.
Liu et al.	Huntington	U	U	U	U	U	U	U	U	U	U	Animal sample size was prospectively estimated (80% power); histological slices were randomly selected, but treatment/group allocation blinding was not identified. Good sample-size reporting, but SYRCLE domains remain mostly unclear.
Segal et al.	MS/EAE	U	U	U	U	U	U	L	L	U	U	Clinical scoring, pain behavior, slide coding, and multiple molecular analyses were blinded; all mice assessed were included in pain analyses. Strong detection-bias reporting and transparent inclusion; selection-bias domains unclear.
Teixeira et al.	MS/EAE	L	U	U	U	U	U	L	U	U	U	Animals were randomly assigned by raffle; experimenter and histological scoring were blinded to group/treatment. Randomization and outcome blinding supported; allocation concealment/random housing unclear.
Campos et al.	PD	L	U	U	U	U	U	U	U	U	U	Rats were randomly divided into sham/stimulation groups; sample size was based on 80% power; seven implant-loss exclusions were reported. Randomization/sample-size reporting is good, but blinding and attrition balance require caution.
Ding et al.	MS/EAE	U	U	U	U	U	U	L	U	U	U	All behavioral tests followed a single-blind principle. Outcome-assessor blinding supported; remaining domains largely unclear.
Sato et al.	PD	L	U	U	U	U	U	U	L	U	U	Animals were randomly assigned; group sizes were kept relatively equal; prespecified ROUT outlier detection/exclusion was reported. Randomization and transparent outcome-data handling supported; blinding not confirmed.
Dalenogare et al.	MS/EAE	U	U	U	U	U	U	L	L	U	U	Operator was blinded to drug administration and genotype; prespecified exclusion criteria were reported and no animals were excluded. Good detection and attrition reporting; randomization/concealment remain unclear.
Sideris-Lampretsas et al.	AD	U	U	U	U	U	U	L	U	U	U	von Frey experimenter was blinded to treatment groups. Outcome-assessor blinding supported; other domains insufficiently reported.
Zhang et al.	PD	U	U	U	U	L	U	L	U	U	U	Investigators were blind to mouse identity in behavioral and experimental procedures. Broad investigator blinding supported; sequence generation and concealment remain unclear.
Godínez-Chaparro et al.	PD	L	U	U	U	U	U	U	U	U	U	Rats were randomly allocated to lesion/control and later treatment groups; immunohistochemical analyses were operator-blinded, but behavioral blinding was not confirmed. Randomization supported; detection bias remains mixed/unclear across outcome types.
Magni et al.	MS/EAE	L	U	U	U	U	U	L	U	U	U	Animals were randomly assigned; motor and trigeminal behavioral measurements were performed by a blinded investigator. Randomization and behavioral detection-bias control supported; other domains unclear.
Wang et al.	PD	L	U	U	U	U	U	L	U	U	U	Mice were randomly assigned; behavioral scorers and data analysts were blinded by coded allocations; intervention/modeling performed by a separate investigator. Randomization and outcome blinding supported; concealment/random housing not clearly described.

Study	Disease	Seq. gen.	Baseline	Alloc. conceal.	Random housing	Blinding caregivers / investigators	Random outcome assess.	Blinding outcome assessor	Incomplete data	Selective reporting	Other bias	Key evidence / interpretation
Cui et al.	PD	U	U	U	U	U	U	U	U	U	U	Full text reviewed. No explicit study-level random sequence generation, allocation concealment, random housing, or blinding was identified; microscopic fields were randomly selected for histological quantification. Predominantly unclear because study-level bias-control procedures were insufficiently reported; random microscopic-field selection does not establish randomized allocation or blinded assessment.
Dworsky-Fried et al.	MS/EAE	L	U	U	U	U	L	L	L	U	U	Male and female C57BL/6 mice were randomly assigned to control or EAE groups. Behavioral testing used prespecified exclusion of animals progressing beyond EAE grade 1. Image acquisition and analysis were blinded to experimental condition and sex; quantified cells were randomly sampled. CPP used a counter-balanced unbiased design. Randomization, random outcome sampling, blinded outcome assessment, and transparent incomplete-outcome handling are supported; allocation concealment, random housing, caregiver/investigator blinding, selective reporting, and other bias remain unclear.
Schmitz et al.	MS/EAE	U	U	U	U	U	U	L	U	U	U	Behavioral nociception was performed without knowledge of genotype; no study-level random allocation wording was identified. Outcome-assessor blinding is supported for nociceptive behavior; other domains remain insufficiently reported.
Giardini et al.	MS/EAE	U	U	U	U	U	U	U	U	U	U	Eligibility is supported by EAE mechanical hyperalgesia together with spinal inflammatory/glial outcomes; bias-control procedures were not sufficiently documented in the preserved extraction. Conservative coding: unreported safeguards remain unclear.
Gao et al.	PD	U	U	U	U	U	U	U	U	U	U	6-OHDA PD rats were assessed for HST/PWT and GFAP-positive astrocytic responses under noradrenergic manipulation; the preserved extraction does not establish randomization or blinding. Conservative coding: core SYRCLE safeguards remain unclear.
Evangelista et al.	MS/EAE	U	U	U	U	L	U	L	U	U	U	The experimenter was blinded to genotype and drug treatment; no explicit study-level randomization wording was identified. Blinding is supported; remaining unreported domains are coded unclear.
Morozzi et al.	MS/EAE	U	U	U	U	U	U	U	U	U	U	Clinical disease score was assessed blinded, but nociceptive-test blinding was not explicitly established; mice reaching EAE score 3.5 were excluded. Blinding applies to clinical scoring only; attrition and other unreported safeguards remain unclear.

SYRCLE coding rule: Low risk was assigned only when the report contained sufficient methodological information. Absence or insufficiency of reporting was coded as Unclear rather than Low. The assessment was performed independently by two investigators, with disagreements resolved by discussion and consensus and, when necessary, consultation with a third investigator.

•
